# Low-temperature culturing improves survival rate of tissue-engineered cardiac cell sheets

**DOI:** 10.1016/j.bbrep.2018.04.001

**Published:** 2018-04-25

**Authors:** Katsuhisa Sakaguchi, Yuto Hinata, Yuki Kagawa, Kiyotaka Iwasaki, Satoshi Tsuneda, Tatsuya Shimizu, Mitsuo Umezu

**Affiliations:** a School of Advanced Science and Engineering, TWIns, Waseda University, 2-2 Wakamatsu-cho, Shinjuku-ku, Tokyo 162-8480, Japan; b Institute of Advanced Biomedical Engineering and Science, TWIns, Tokyo Women's Medical University, 8-1 Kawada-cho, Shinjuku-ku, Tokyo 162-8666, Japan; c Ogino Memorial Laboratory, Nihon Kohden Corporation, TWIns, 8-1 Kawada-cho, Shinjuku-ku, Tokyo 162-8666, Japan

**Keywords:** Cell sheet, Low-temperature culture, Three-dimensional tissue, Vascularization

## Abstract

Assembling three-dimensional (3D) tissues from single cells necessitates the use of various advanced technological methods because higher-density tissues require numerous complex capillary structures to supply sufficient oxygen and nutrients. Accordingly, creating healthy culture conditions to support 3D cardiac tissues requires an appropriate balance between the supplied nutrients and cell metabolism. The objective of this study was to develop a simple and efficient method for low-temperature cultivation (< 37 °C) that decreases cell metabolism for facilitating the buildup of 3D cardiac tissues. We created 3D cardiac tissues using cell sheet technology and analyzed the viability of the cardiac cells in low-temperature environments. To determine a method that would allow thicker 3D tissues to survive, we investigated the cardiac tissue viability under low-temperature culture processes at 20–33.5 °C and compared it with the viability under the standard culture process at 37 °C. Our results indicated that the standard culture process at 37 °C was unable to support higher-density myocardial tissue; however, low-temperature culture conditions maintained dense myocardial tissue and prevascularization. To investigate the efficiency of transplantation, layered cell sheets produced by the low-temperature culture process were also transplanted under the skin of nude rats. Cardiac tissue cultured at 30 °C developed denser prevascular networks than the tissue cultured at the standard temperature. Our novel findings indicate that the low-temperature process is effective for fabricating 3D tissues from high-functioning cells such as heart cells. This method should make major contributions to future clinical applications and to the field of organ engineering.

## Highlights

• Low-temperature culture conditions maintained layered myocardial cell sheets.
• Cardiac tissue cultured at 30 °C developed denser prevascular networks than the 37 °C cultivation.
• Low-temperature tissue engineering could possibily improve cell delivery for transplantation.

## 1 Introduction

In the near future, three-dimensional (3D) tissue engineering is expected to be applied to evaluations of drug efficiency, with eventual application to medical treatments to replace organ transplantation [1]. However, a crucial issue with respect to tissue and organ engineering is how to create vessels that function as capillaries, veins, and arteries, allowing blood to supply nutrients and oxygen as well as to remove waste products [2]. Many laboratories have developed cell delivery systems and bioreactors that can produce hydro-pressure and a flow of medium with shear stress for maintaining endothelial cell networks in high-density engineered tissues [3], [4], [5]. Our laboratory has successfully produced cardiac tissues with perfusable vascular networks by layering cell sheets cultured on a collagen bed with microchannels and a vascular bed from rat legs [6], [7]. This process of layering cell sheets, called “cell sheet engineering,” has been used with several different cell types [8], [9], [10], [11]. Cell sheet engineering technology has the potential to create various functional cell-dense tissues for treating damaged tissues and organs [12]. However, problems with engineering a functional thicker tissue are expected based on similar issues observed in organs currently preserved by the latest technology [13]. Cardiomyocytes, hepatocytes, and neurocytes have a higher metabolic rate and produce more waste molecules than other cells. Therefore, for their culture, it is necessary for the medium to have a functional ability matching that of real blood to neutralize waste molecules under oxidative stress during processes of energy production [14], [15]. In this study, we report the development of a new strategy using a low-temperature culture process at 20–33.5 °C that (1) inhibited the production of waste molecules and (2) maintained 3D cardiac tissues, produced by cell sheet technology, in good condition. Furthermore, prevascular network formation was observed under the same low-temperature culture conditions. Previous studies have shown that endothelial cells naturally form a prevascular network at the physiological temperature of 37 °C [16]. Because low-temperature cultivation could also spontaneously induce the formation of prevascular networks, thicker cardiac tissues with prevascular networks can be produced for transplantation. Therefore, this study also examined whether the proposed low-temperature cultivation could effectively and efficiently produce thicker-layered cell sheet tissues with the necessary prevascular network for applications in tissue engineering and regenerative medicine.

## 2 Materials and methods

All animal experiments were performed according to the “Guidelines of Waseda University and Tokyo Women's Medical University on Animal Use.”

### 2.1 Preparation of neonatal rat cardiac cell sheets

Cardiomyocytes were isolated from the ventricular hearts of 1-day-old Sprague Dawley (SD) rats (Harlan Laboratories Japan, Tokyo, Japan) using Hanks balanced salt solution (H9394; Sigma Aldrich, St. Louis, MO, USA) with 0.5 mg/mL collagenase type 2 (4176; Worthington Biochemical Corporation, Lakewood, NJ, USA), as previously described [17]. Cell suspensions were seeded at a density of 3.6 × 10^6^ cells per dish on a temperature-responsive cell culture dish (UpCell, Ø 35 mm, Type-E; CellSeed, Tokyo, Japan) precoated with 1 mL of fetal bovine serum for 3 days. After seeding the cardiomyocytes, the dish was incubated at 37 °C for 4 days. Confluent cells were harvested as a uniform single, intact, and viable cell sheet after incubating the culture dish in a CO_2_ incubator set at 20 °C for 20 min. The harvested cell sheet had a diameter of approximately 15 mm and a thickness of 10 µm.

### 2.2 Viability measurement

To count the number of living cells, the cultured cell sheet was rinsed with 0.5 mg/mL collagenase medium for isolating the cells, which were stained with trypan blue (T8154; Sigma Aldrich). To determine the metabolism of the cardiomyocytes, the consumption of glucose, the production of lactic acid, and the quantity of lactase dehydrogenase (LDH) leakage were measured by an independent outsourcing laboratory (SRL, Tokyo, Japan). The oxygen concentrations were also measured to confirm the decrease of metabolism under the low-temperature conditions (LTCs), using a Clark-type oxygen micro-sensor (OX-50; Unisense, Denmark), at intervals of 100 µm in terms of the distance from the culture dish bottom. The details of this measurement system and calculation method were described previously [18].

### 2.3 Histological analysis

After cultivation under various conditions, cell sheets were fixed with 4% paraformaldehyde (Wako Pure Chemicals, Osaka, Japan) and routinely processed into 5-μm-thick paraffin-embedded sections, which were stained with hematoxylin and eosin by conventional methods and observed under a microscope (ECLIPSE E800; Nikon, Tokyo, Japan). To detect cardiomyocytes, the specimens in the cell sheet were immunolabeled with an anti-troponin T primary antibody (Cardiac Isoform Ab-1, Clone: 13-11; Thermo Scientific, Waltham, MA, USA) at a dilution of 1/100 for 2 h at room temperature, followed by immunolabeling with an Alexa Fluor 488-conjugated anti-mouse IgG secondary antibody (A11017; Invitrogen, Carlsbad, CA, USA) at a dilution of 1/200 for 2 h at room temperature. Cells were then observed under a fluorescence microscope. After staining for troponin T, images were also analyzed using Image J software to quantify the area of the transplanted cardiac tissue.

### 2.4 Prevascularization of endothelial cells in cardiac cell sheets

To observe their prevascular network formation, collected endothelial cells were replaced with green fluorescent protein (GFP)-expressing endothelial cells by magnetic cell sorting (MACS), as described previously [19]. To separate endothelial cells from normal SD rats and GFP-expressing SD rats (Sankyo Lab Service, Hamamatsu, Japan), the suspension culture medium with primary myocardial cells was incubated with a mouse monoclonal anti-rat CD31 antibody (MCA1334G; AbD Serotec, Kidlington, UK) for 30 min at room temperature. After being washed with running buffer medium [phosphate-buffered saline (PBS) containing 5% bovine serum albumin and 2 mmol/L ethylenediaminetetraacetic acid], cells were incubated with anti-mouse IgG-conjugated microbeads (130-048-401; Miltenyi Biotec, Bergisch Gladbach, Germany) for 15 min at room temperature. After incubation, the cells were washed again with running buffer medium. The suspension containing the cells attached to the microbeads was allowed to pass through an LS column (130-042-401; Miltenyi Biotec) in a magnetic field generated by a cell sorter system (130-042-501, MiniMACS System; Miltenyi Biotec). The LS column was then washed with running buffer medium to remove the magnetic fields from the system, and the cells trapped inside the LS column were flushed out with running buffer medium. To produce myocardial cell sheets containing GFP-positive endothelial cells, GFP-negative cardiac cells (without normal endothelial cells) and purified GFP-positive endothelial cells were mixed at a ratio of 7:1.

### 2.5 Analysis of endothelial cell vascular networks

Cell sheets containing GFP-expressing endothelial cells were photographed with a fluorescence microscope. The length of the prevascular network in each cell sheet was measured with imaging analysis software (Angio Tool; National Cancer Institute, Bethesda, MD, USA).

### 2.6 Flow cytometry

To analyze the populations of endothelial cells and cardiomyocytes in a given cell sheet, flow cytometry was performed using a Gallios cytometer (Beckman Coulter, CA, USA). The suspension containing the isolated cells was incubated with a mouse monoclonal anti-rat CD31 antibody (MCA1334G; AbD Serotec) at a dilution of 1/100 for 30 min at room temperature; cardiomyocytes were incubated with an anti-troponin T antibody (Cardiac Isoform Ab-1, Clone: 13-11; Thermo Scientific, Waltham, MA, USA) at a dilution of 1/100 for 30 min at room temperature. After being washed with PBS, cells were incubated with PE Goat anti-mouse IgG antibody (405307; BioLegend, CA, USA) at a dilution of 1/100 for 30 min at room temperature. After the antibody reaction, the cells that reacted were analyzed.

### 2.7 Transplantation of cell sheets

The low-temperature-processed and standard quintuple-layered cell sheets were transplanted under the skin on the back of nude rats (F344/NJcl-rnu/rnu, 8–12 week-old males; CLEA Japan, Tokyo, Japan). For the procedure, nude rats were anesthetized using 2% isoflurane. After the subcutaneous tissue was opened to a 2–3 cm square, the cell sheet was placed under the skin on the back and covered with a silicone sheet (0.5 mm in thickness). At 2 weeks after transplantation, the transplanted cell sheet was removed and treated for observation (*n* = 3 per group, with and without low-temperature treatment).

### 2.8 Electric potential measurement

Two weeks after transplanting the cell sheet, the transplanted place was opened and the pulsation of the transplanted cell sheet was confirmed. After confirming the contraction, the electric potential was measured by inserting the electrodes at two end portions of the graft (ER-1, Cygnus Technology, NC, USA). The acquired potential was collected and analyzed using a data acquisition system (PowerLab, ADInstruments, New South Wales, Australia).

## 3 Results

### 3.1 Viability of cardiac cell sheets produced under low-temperature conditions

**Fig. 1:**
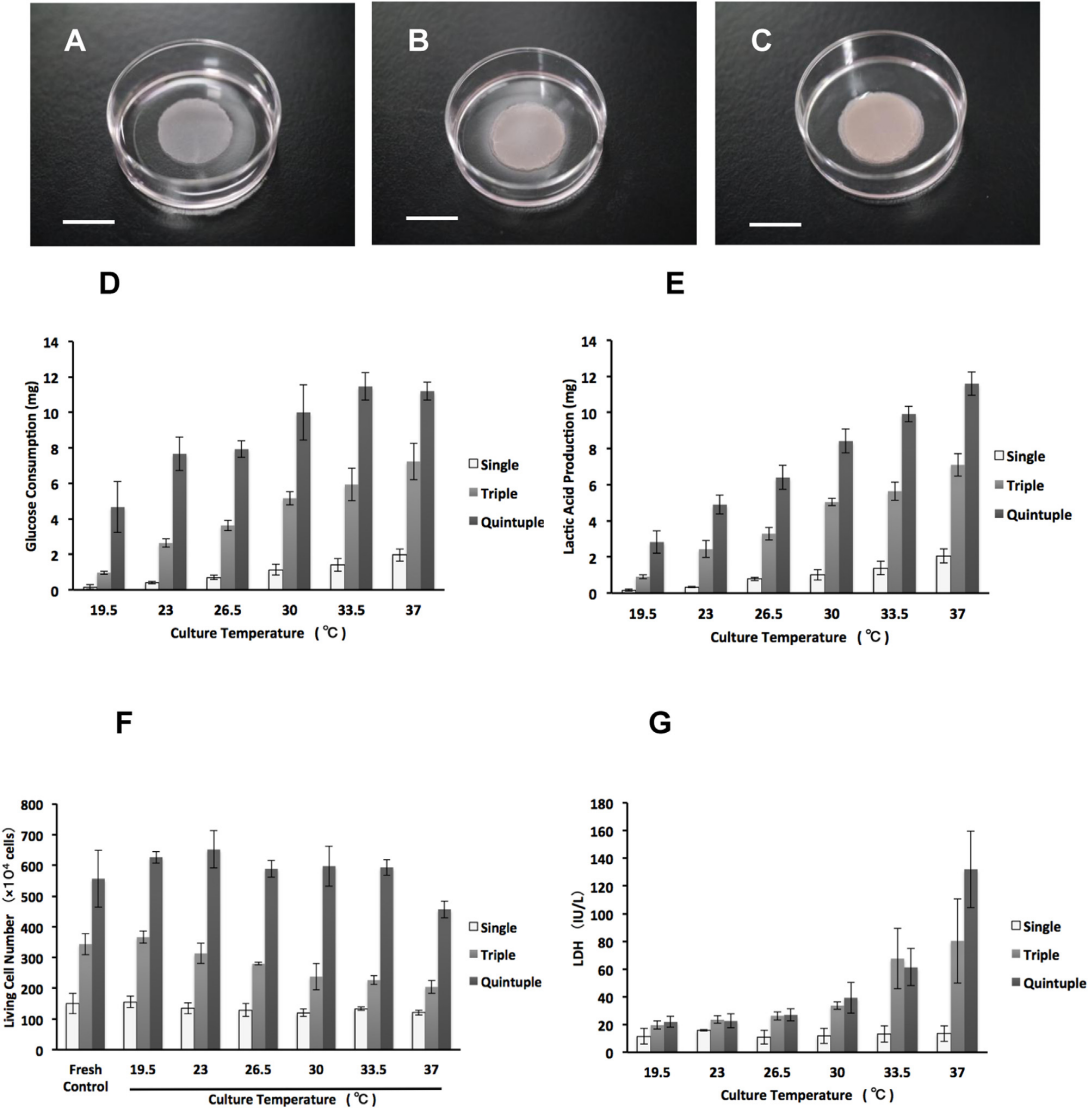
**Viability of layered cell sheets produced by the low-temperature process.** Photographs A–C show single-, triple-, and quintuple-layered cardiac cell sheets on culture dishes, respectively. (A) A single-layered cell sheet, prepared from neonatal rat cardiac cells, was allowed to detach from a temperature-responsive culture dish and transferred to another dish for removing culture medium. (B) A triple-layered cell sheet and (C) a quintuple-layered cell sheet (scale bars, 10 mm). (D) Temperature-dependent glucose consumption of multilayered cell sheets after 3 days of cultivation. (E) Lactic acid production of multilayered cell sheets. Glucose consumption and lactic acid production decreased with decreasing temperature, indicating that low-temperature caused a decrease in cellular activity. (F) Numbers of living cells stained by trypan blue. (G) The numbers of dead cells were calculated by measuring lactate dehydrogenase after 3 days of cultivation at various low temperatures. These results show that single-layered cell sheets survived at all temperatures; however, in triple- and quintuple-layered cell sheets, dead cells were found to increase at temperatures greater than 33.5 °C.

Cardiomyocytes were isolated from neonatal rat hearts and used to prepare sheet-shaped cardiomyocytes on a thermo-responsive culture dish (Ø 35 mm). Single-, triple-, and quintuple-layered cell sheets were removed from the surface of the dish using a careful pipet maneuver described previously [20]. Cell sheets were cultured on normal culture dishes (Ø 35 mm) at 19.5 °C, 23 °C, 26.5 °C, 30 °C, 33.5 °C, and 37 °C for 3 days (Fig. 1A–C) with 2 mL of culture medium. The detached layered cell sheets were observed to have shrunk, exhibiting a diameter of approximately 15 mm. After 3 days of cultivation, the consumption of glucose and production of lactic acid were measured at various temperatures between 19.5 °C and 37 °C (Fig. 1D and E). Our results showed that glucose consumption and lactic acid production decreased with decreasing temperature. Moreover, to confirm decreases in the cell activity and metabolism under LTCs, the oxygen concentrations of culture media were measured on the single-layer cell sheet at two temperatures: 30 °C and 37 °C, by a Clark-type oxygen microneedle sensor. The gradients of the oxygen concentration graphs at 30 °C and 37 °C were 1.27 ± 0.06 and 1.61 ± 0.06 mg/L/mm, respectively. The gradient of the oxygen concentration in the 37 °C cultivation was steeper than that in the 30 °C cultivation (Fig. S1A). The calculated oxygen consumption rates at 30 °C and 37 °C were 0.16 ± 0.001 and 0.27 ± 0.001 pmol/h/cell, respectively, with a significant difference between them (Fig. S1B). These results show that the low-temperature process caused decreases in cell activity and metabolism. The numbers of dead and living cells were also measured to investigate the effect of the low-temperature treatment on cell activity. After 3 days of cultivation, the numbers of living cells stained with trypan blue were counted (Fig. 1F). In addition to the living cell analysis, the numbers of dead cells were estimated by measuring the enzymatic activity of LDH (Fig. 1G). LDH normally exists inside the cellular membrane; however, the cellular membrane typically ruptures at the time of cell death and releases LDH. Our results demonstrated that single-layered cell sheets were able to survive at all temperatures, whereas triple- and quintuple-layered cell sheets were unable to survive at temperatures greater than 33.5 °C. These findings indicated that (i) a high metabolic rate could reduce the viability of cardiomyocytes and (ii) a low metabolic rate induced from the low culturing temperature could enhance the survival rate.

### 3.2 Histological analysis of layered cardiac cell sheets after low-temperature cultivation

**Fig. 2:**
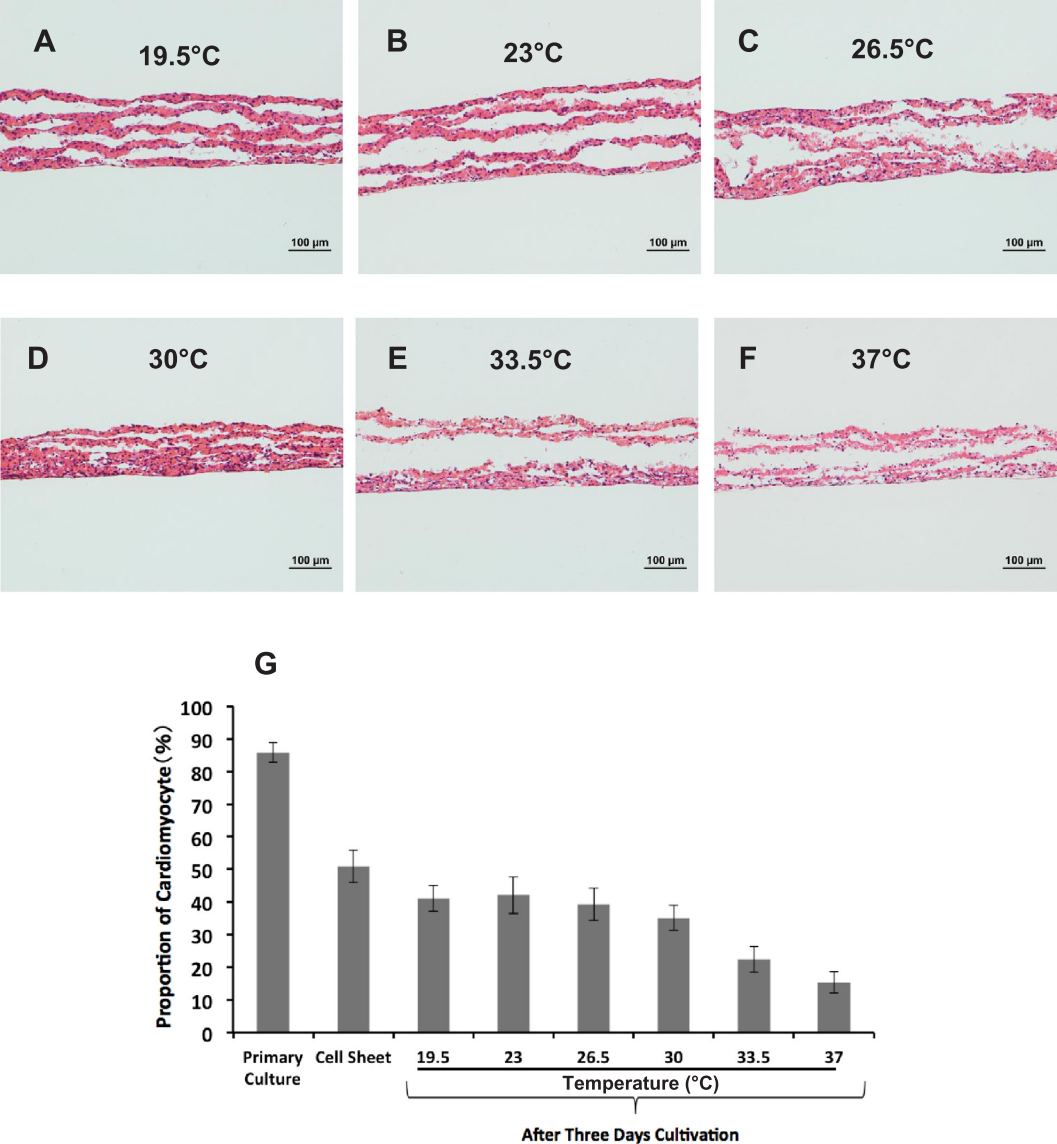
**Histological analysis of multilayered cell sheets under low-temperature conditions.** Hematoxylin and eosin-stained sections of quintuple-layered cardiac cell sheets cultured for 3 days at various temperatures: (A) 19.5 °C, (B) 23 °C, (C) 26.5 °C, (D) 30 °C, (E) 33.5 °C, and (F) 37 °C. Cultivation at 19.5 °C and 23.5 °C produced large gaps among the cell layers in cell sheets; at 33.5 °C and 37 °C, the cells lost their nuclei (scale bar, 100 µm). (G) Flow cytometry was performed to detect cardiomyocytes. As a result, approximately 86% of the cells were cardiomyocytes in the primary culture shortly after collection from the eviscerated heart of neonatal rats. After 4 days of cultivation for fabricating cell sheets, the proportion of cardiomyocytes in the cell sheet decreased to approximately 51%. After 3 days of cultivation at various temperatures, the proportion of cardiomyocytes in cell sheets was measured. Comparable results were obtained from histological analysis, which showed that the low-temperature culture condition was able to maintain a cardiomyocyte rate of ~ 40% at temperatures up to 30 °C. However, at temperatures of 33.5 °C and 37 °C, the cardiomyocyte rates decreased to ~ 22% and ~ 15%, respectively.

Layered cell sheets incubated at low temperatures were subjected to histological analysis. The specimens of single-, triple-, and quintuple-layered cell sheets were stained with hematoxylin–eosin. Fig. 2A–F shows the specimens of quintuple-layered cell sheets cultured at 19.5–37 °C. The microphotographs of single- and triple-layered cell sheets are shown in the Supplemental figures (Fig. S2 and S3). The microphotographs of single- and triple-layered cell sheets demonstrated that (i) the layered cell sheets became thinner with increasing cultivation temperature and (ii) the cells had higher cellular activity and spread out at higher cultivation temperatures. However, in the case of the quintuple-layered cell sheet, gaps between the cell sheets were observed and many of the nuclei could not be observed at higher cultivation temperatures. To observe cardiac muscle cells, the specimens of single-, triple-, and quintuple-layered cell sheets were stained with troponin T (Fig. S4–6). The microphotographs demonstrated that the intensity of the green color, indicating cardiac muscle cells, decreased with increasing temperature, and this color was hardly detectable above 33.5 °C. To measure the proportion of cardiomyocytes, the cells in the sheets were analyzed using flow cytometry (Fig. 2G). Approximately 86% of the cells were cardiomyocytes in the primary culture shortly after collection from the eviscerated hearts of neonatal rats. After 4 days of cultivation to produce a single cell sheet, the proportion of cardiomyocytes in the cell sheet decreased to approximately 51%. After 3 days of cultivation on normal dishes at various temperatures, the proportions of cardiomyocytes in the single cell sheet were measured again. The low-temperature culture condition was able to maintain the proportion of cardiomyocytes at ~ 40% at a temperature of up to 30 °C. These results were comparable with those obtained from the histological analysis. However, at 33.5 °C and 37 °C, the proportions of cardiomyocytes decreased to ~ 22% and ~ 15%, respectively, indicating that at temperatures greater than 33.5 °C, the high metabolic rate reduced cardiomyocyte viability.

### 3.3 Development of prevascular networks under low-temperature conditions

**Fig. 3:**
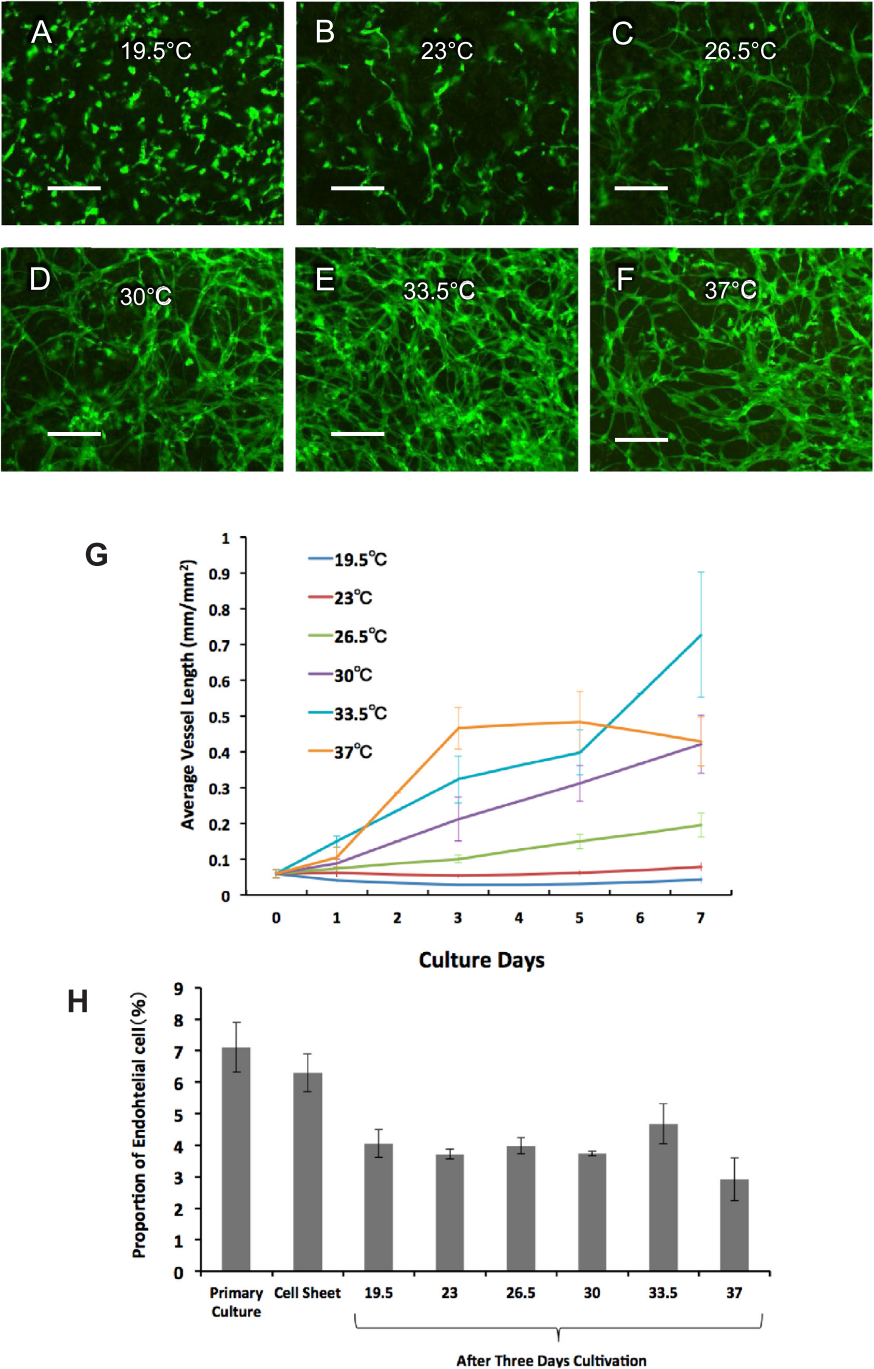
**Endothelial cell function in low-temperature conditions.** The development of vascular networks in endothelial cells under low-temperature conditions was observed. To observe vascular formation under a microscope in real time, endothelial cells were replaced with green fluorescent protein (GFP)-positive endothelial cells using a magnetic cell sorting method. Cardiomyocytes with GFP-positive endothelial cells were seeded to culture dishes and cultured for 7 days at temperatures of (A) 19.5 °C, (B) 23 °C, (C) 26.5 °C, (D) 30 °C, (E) 33.5 °C, and (F) 37 °C. (E) The most developed vascular network was found upon cultivation at 33.5 °C (scale bars, 100 µm). (G) Lengths of vascular networks in endothelial cells were measured on the microphotographs of cells by imaging analysis software at various temperatures. After 3 days of cultivation, vascularization developed more rapidly at higher temperatures. However, the development of the network stagnated after 5 days at 37 °C. On the other hand, cultivation at 33.5 °C produced more developed vascularization at 7 days. (H) Flow cytometry for detecting endothelial cells in primary cardiac cells was performed. The results showed that the number of endothelial cells was reduced after 7 days of cultivation at 37 °C; these results were consistent with those from the network length analysis.

As described above, although low-temperature cultivation maintained the viability of cardiomyocytes, a prevascular network still needed to be created within the engineered cardiac tissues to ensure that the transplantation of the cell sheet to the target site would be successful [21]. Therefore, we also investigated the development of prevascular networks in cardiac cell sheets under the low-temperature cultivation conditions. To observe prevascular formation under a microscope in real time, endothelial cells were replaced with GFP-positive endothelial cells by MACS. Cardiomyocytes containing GFP-positive endothelial cells were seeded onto culture dishes and cultured at 19.5–37 °C for 7 days (Fig. 3A–F). Furthermore, the length of the vascular network was measured by imaging analysis (Fig. 3G). After 3 days of cultivation, the development of prevascularization was enhanced by increasing the temperature. Although the development of a network was found to stagnate after 5 days of cultivation at 37 °C, it was enhanced by 7 days of cultivation at 33.5 °C. To measure the population of CD31-positive cells (endothelial cells) in the cell sheet condition, flow cytometry was also performed. The results showed that the population of endothelial cells was reduced in the 3 days of cultivation at 37 °C (Fig. 3H), which was consistent with the analysis of network length. These results indicated that cultivation at 30–33.5 °C was appropriate to create a prevascular network in cardiomyocyte sheets. In contrast, cultivation at 37 °C decreased not only the viability of cardiomyocytes but also in the population of endothelial cells.

### 3.4 Transplantation of vascularized multilayered cell sheets prepared under low-temperature conditions

**Fig. 4:**
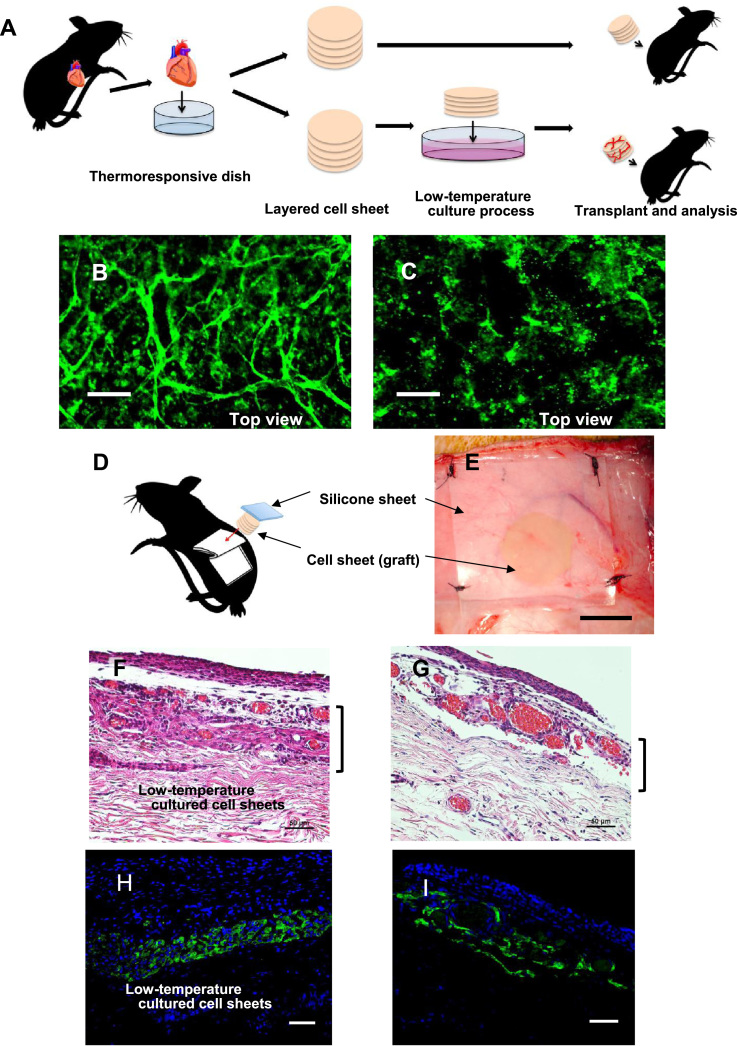
**Implantation of quintuple-layered cell sheets into nude rats.** (A) Schematic illustration of the implantation of quintuple-layered cell sheets into nude rats. (B) The top view microphotograph of a CD31-stained quintuple-layered cell sheet, which was cultured at 30 °C for 7 days to produce a vascular network (scale bar, 100 µm). (C) The top view microphotograph of a CD31-stained, freshly prepared quintuple-layered cell sheet. The former cell sheet, which was obtained by cultivation at 30 °C, shows dense vascular networks (scale bar, 100 µm). (D) Schematic illustration shows the transplanted site of a quintuple-layered cell sheet. (E) Macro-photograph of a transplanted cell sheet on the tissue of a rat (scale bar, 10 mm). At 2 weeks after transplantation, the transplanted cell sheet was removed from the nude rat. The specimens were stained with hematoxylin–eosin for histological analysis (F, G) (scale bars, 50 µm). The low-temperature-treated quintuple-layered cell sheet (F) was found to be engrafted more clearly than the standard cell sheet (G), and the remaining cardiomyocytes were confirmed by troponin T staining (H, I) (scale bars, 50 µm).

Low-temperature cultivation (< 30 °C) maintained the viability of cardiomyocytes and prevascular networks developed in the cell sheets at temperatures up to 30 °C. Therefore, we also examined whether a prevascularized quintuple-layered cell sheet prepared by the low-temperature cultivation method would produce more effective tissue transplantation outcomes than a quintuple-layered cell sheet prepared by the standard process. In the standard process, a quintuple-layered cell sheet was processed at 37 °C and was immediately transplanted into a rat after its detachment from a culture dish. Fig. 4A shows a strategy for investigating the efficiency of the low-temperature culture process. First, after 7 days of low-temperature cultivation, the prevascularization of quintuple-layered cell sheets was observed. Although the top view of a CD31-stained quintuple-layered cell sheet cultured at 30 °C indicated the successful creation of a viable prevascular network after 7 days (Fig. 4B), the CD31-stained quintuple-layered cell sheet prepared through the standard process created a poor prevascular network (Fig. 4C). These results showed that the low-temperature-processed cell sheet was able to create denser prevascular networks than the standard cell sheet.

To investigate the function of this prevascularization, low-temperature- and standard-processed quintuple-layered cell sheets were transplanted under the skin on the back of nude rats. Fig. 4D and E shows an illustration of the cell sheet transplantation site and a macroscopic photograph of the transplantation site, respectively. The cell sheets were placed under the skin on the back and covered with a silicone sheet (0.5 mm in thickness). At 2 weeks after transplantation, the transplanted low-temperature cell sheet yielded denser vascularization and produced stronger pulsating power than the standard sheets (Supplemental Movie). After observation, the transplanted cell sheet was removed from the nude rat and routinely processed to produce 10 µm specimens, which were stained with hematoxylin–eosin for histological analysis. The low-temperature-treated quintuple-layered cell sheet (Fig. 4F) was found to be engrafted more clearly than the standard cell sheet (Fig. 4G). Fig. 4H shows the low-temperature-treated quintuple-layered cell sheet stained with troponin T (green). Fig. 4I shows the stained section without the treatment. The remaining cardiomyocytes in the low-temperature sheet were confirmed to be denser than those in the regular sheet by troponin T staining. The remaining cardiomyocytes were then measured and quantified by image analysis. Quantitative analysis of troponin-positive tissues demonstrated the survival of cardiac tissue treated under LTCs [(−) LTC: 8302 ± 2700 µm versus (+) LTC: 11,530 ± 4550; Fig. S7]. Based on this analysis, no significant differences were found between with and without the low-temperature treatment. However, the findings indicated that the low-temperature treatment could possibily improve cell delivery. Furthermore, the low-temperature-processed cell sheet contained many sarcomere structures (Fig. S8A). In contrast, no sarcomere structures were observed in the standard sheet (Fig. S8B). The electric potential of the transplanted cell sheets was also measured, and the potential differences were observed to originate from the host's breathing and heartbeat (Fig. S9). The transplanted cell sheet was confirmed to be firmly engrafted with preserved function, indicating that the prevascularized cell sheet prepared by the low-temperature process provided more secure cell transplantation than that prepared by the standard process.

Supplementary material related to this article can be found online at doi:10.1016/j.bbrep.2018.04.001.

The following is the Supplementary material related to this article Video 1.

Supplementary Video 1 : "Pulsating tissue composed of five transplanted myocardial cell sheets".

## 4 Discussion

The most appropriate temperature for cardiac tissue engineering has been thought to be similar to that in the in vivo situation, namely, 37 °C, which has been used for developing prevascular networks, growing cells, and maintaining their functions. However, in an *in vivo* situation, other organs have to preserve stable blood functions for supplying nutrients and oxygen and removing waste products. Regular cell culture medium cannot adequately function to maintain cardiac tissue because a temperature of 37 °C is unsuitable for heart muscle cells with such a high metabolic rate. Functional cells such as hepatocytes, cardiomyocytes, and neurons require a large amount of energy and contain a larger number of mitochondria than other cells. Indeed, cell membranes suffer oxidative stress from the large energy production [22]. Thus, *in vitro* conditions also need rapid neutralization measures, including antioxidants to handle oxidative stress, similar to the neutralization measures naturally found in the body. However, regular culture medium has no ability to neutralize waste products [14]. In this study, however, we report that lowering the cell culture temperature decreased the metabolic rate of cardiomyocytes, which in turn reduced the level of waste products and conferred a more appropriate environment for tissue engineering. Indeed, severe necrosis in heart muscle cells was observed at the normal culture temperature. In contrast, by lowering the cell culture temperature, a substantial improvement in the survival rate of heart muscle cells was observed.

A key factor for tissue engineering is the development of prevascular networks. Previous studies indicated that prevascularization is very important for cell sheet transplantation [16], [19], [23]. Prevascular networks have been confirmed to develop at temperatures higher than 26.5 °C. Low-temperature vascularization is a common and naturally occurring phenomenon in the body surface and can be easily observed when skin wounds heal at a low-temperature, such as in the earlobe at 29 °C [24]. In this study, a prevascularized quintuple-layered cell sheet prepared by the low-temperature process was transplanted into a nude rat and was able to maintain its cardiomyocyte viability at the site more effectively than sheets prepared by the standard process. The findings of efficient transplantation observed in this study were due to the stable connections of the prevascular network among the cell layers in the cell sheet. Quintuple-layered cell sheets prepared by the standard process showed no obvious vascular network connections among the cell layers, and the host blood supply was unable to flow into the transplanted cell sheets, which resulted in cell sheet necrosis. In fact, the standard cell sheets produced blood clots that were observed in the transplanted cell sheet 1 day after transplantation; these blocked the blood flow and produced an irregular shape in the transplanted tissue.

Currently, tissues with high metabolism, such as the heart, liver, and kidney, cannot maintain their normal function in standard cultivation. However, using the low-temperature cultivation method described here, we showed that prevascular networks in multilayered cell sheets developed and were used to maintain the function of tissue with high metabolism, which was then successfully transplanted. This new method should provide a novel approach for developing more effective organ engineering applications in the future.
