# Supplementary material for: Low-temperature culturing improves survival rate of tissue-engineered cardiac cell sheets
Source: Biochem Biophys Rep. 2018 Apr 25;14:89–97. doi: 10.1016/j.bbrep.2018.04.001 (PMC5986703; doi:10.1016/j.bbrep.2018.04.001)
Supplement: Supplementary file 2 — Supplementary material [file mmc1.docx]

**Conflicts of interest**

Tatsuya Shimizu is a scientific advisory board member and a stakeholder of CellSeed Inc. Tokyo Women's Medical University receives research funds from CellSeed Inc.
