# Supplementary material for: Low-temperature culturing improves survival rate of tissue-engineered cardiac cell sheets
Source: Biochem Biophys Rep. 2018 Apr 25;14:89–97. doi: 10.1016/j.bbrep.2018.04.001 (PMC5986703; doi:10.1016/j.bbrep.2018.04.001)
Supplement: Supplementary file 3 — Supplementary material [file mmc2.docx]

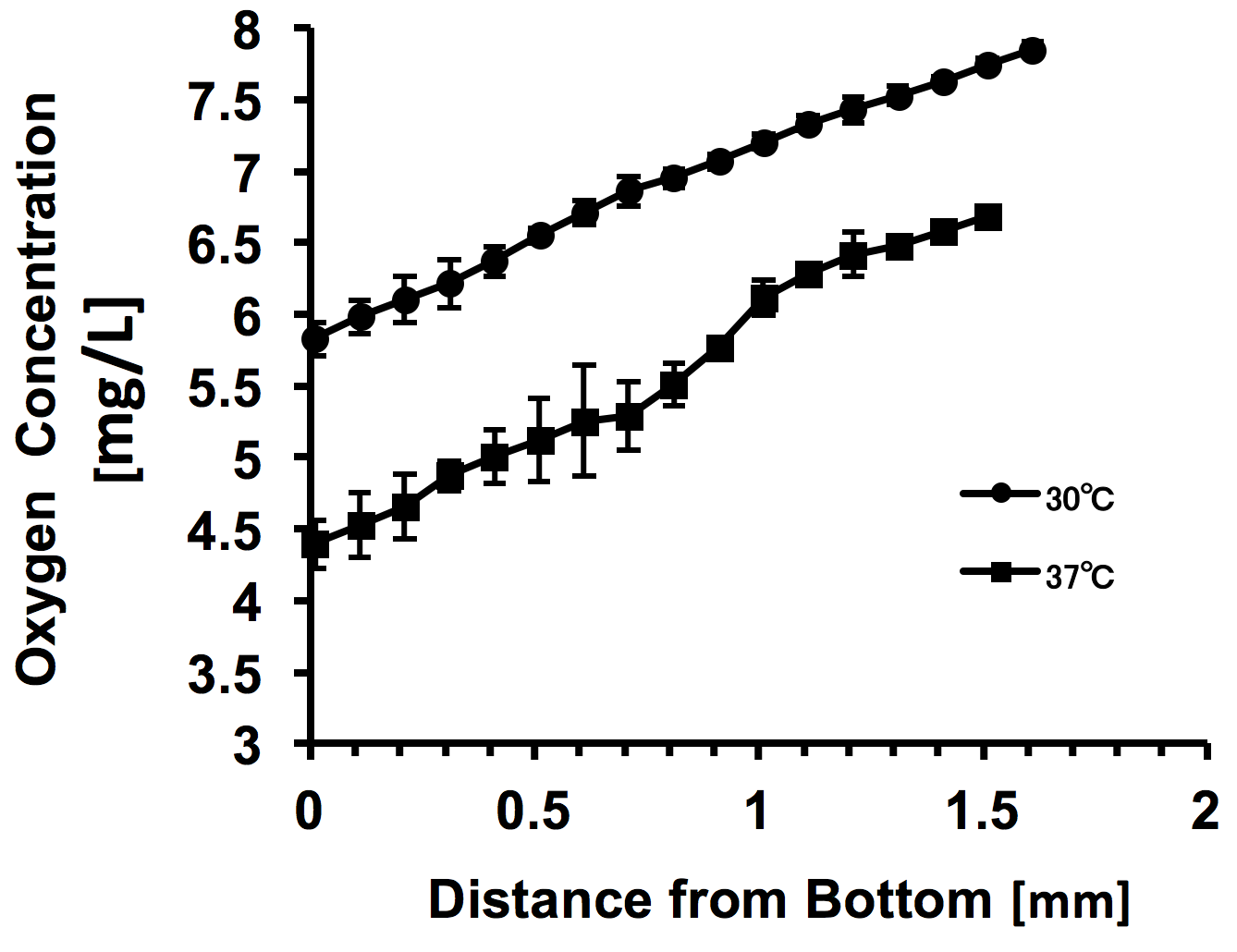

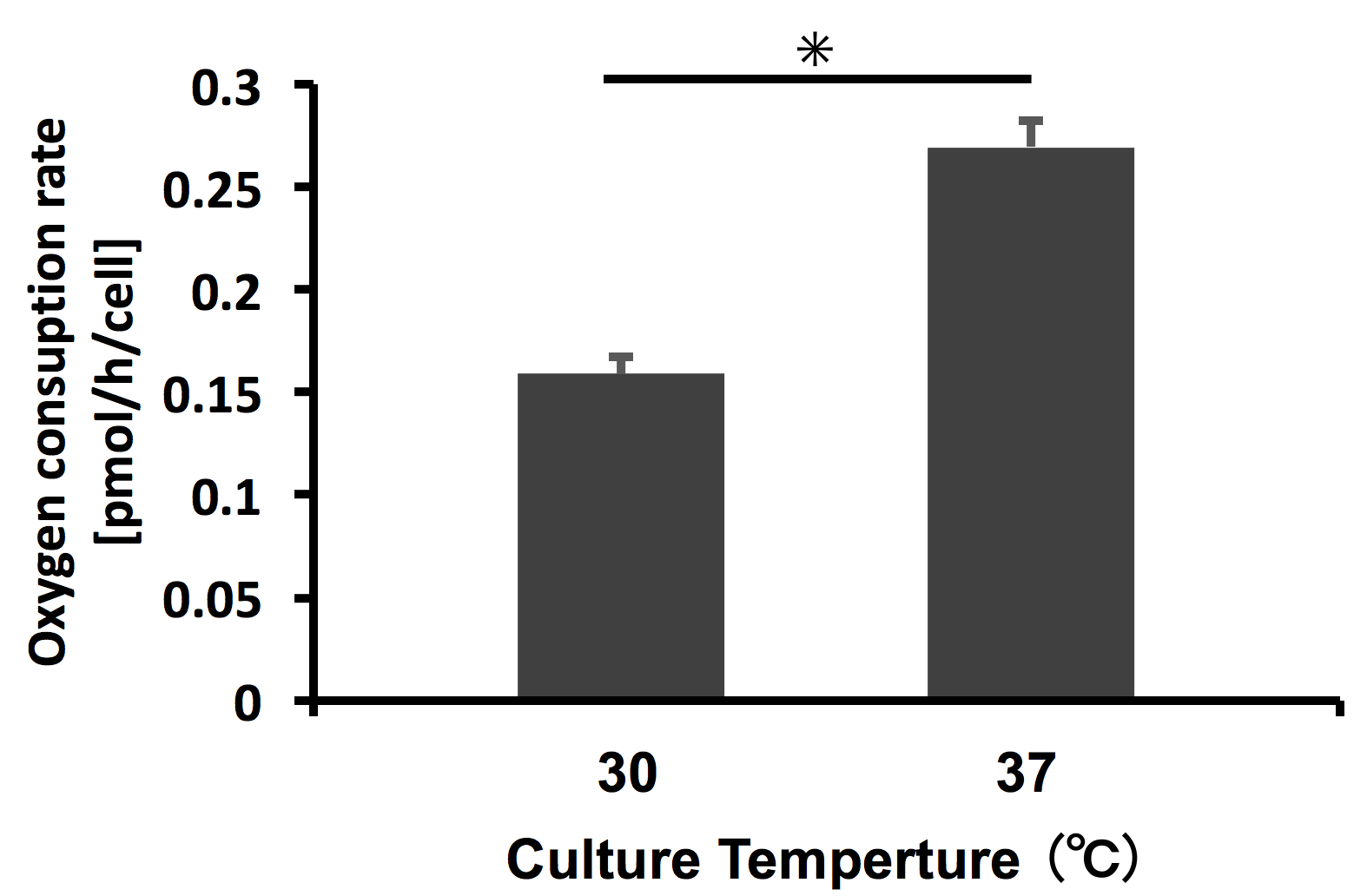


**A**

**B**

Figure S1. The measurement of oxygen concentration and calculation of oxygen consumption of cardiomyocytes after 3 days of cultivation at two temperatures: 30°C and 37°C. (A) The oxygen concentrations were measured at intervals of 100 μm in terms of the distance from the culture dish bottom [n = 3]. Error bars represent the SD. (B) The average oxygen consumption rate at each culture temperature, 30°C and 37°C, is shown [n = 3]. Error bars represent the SD. Asterisk shows a significant difference based on the *t-*test (p < 0.05).


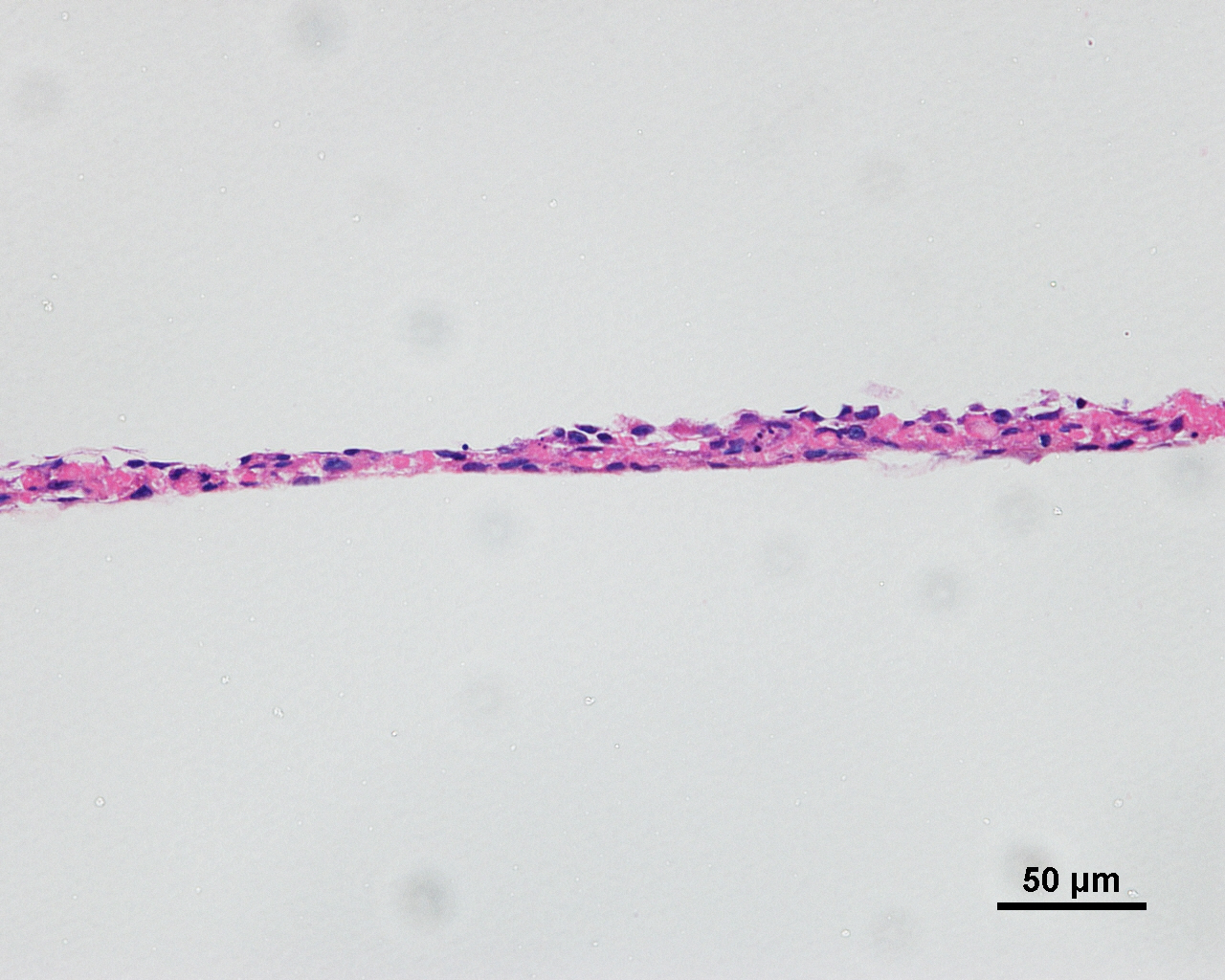

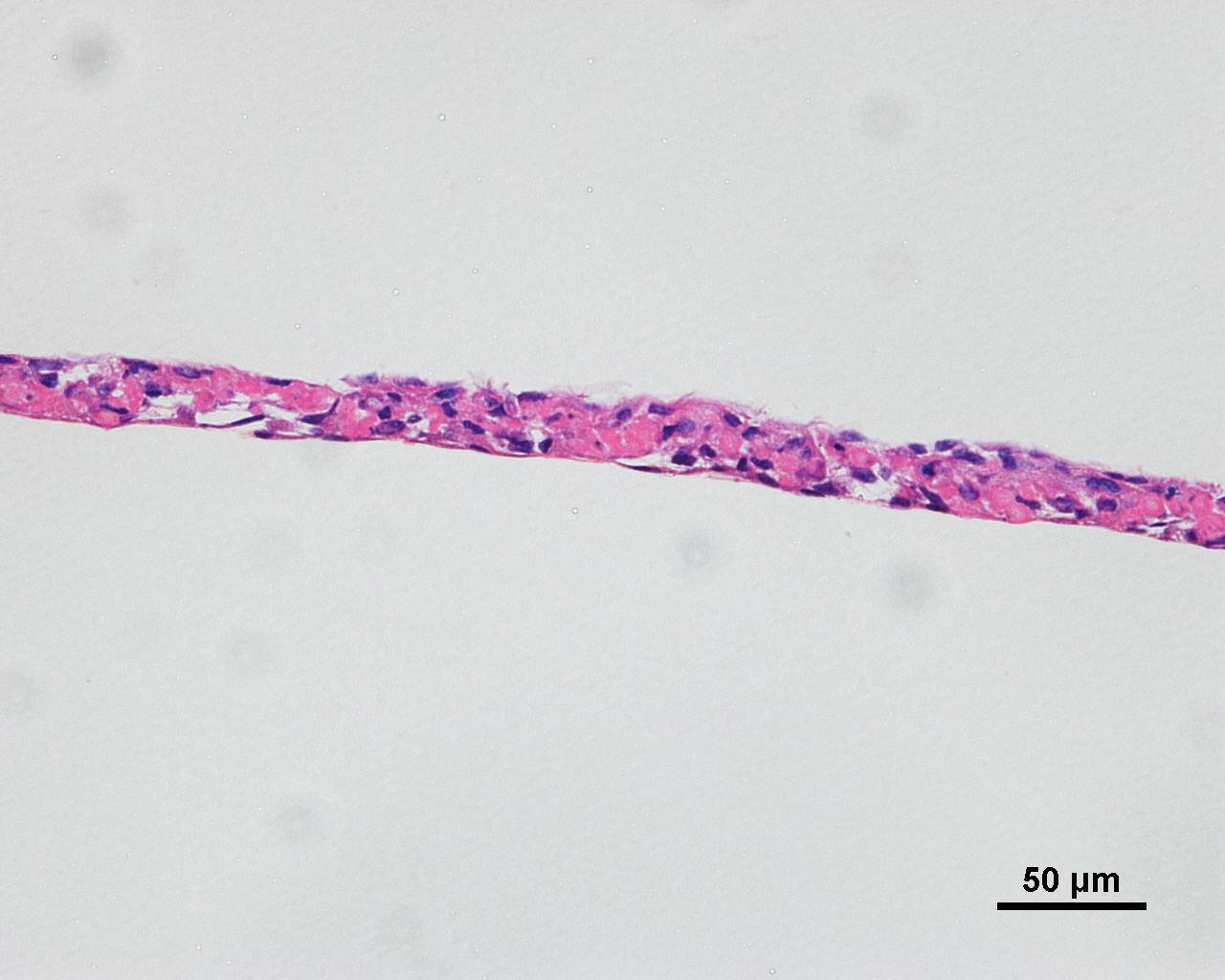

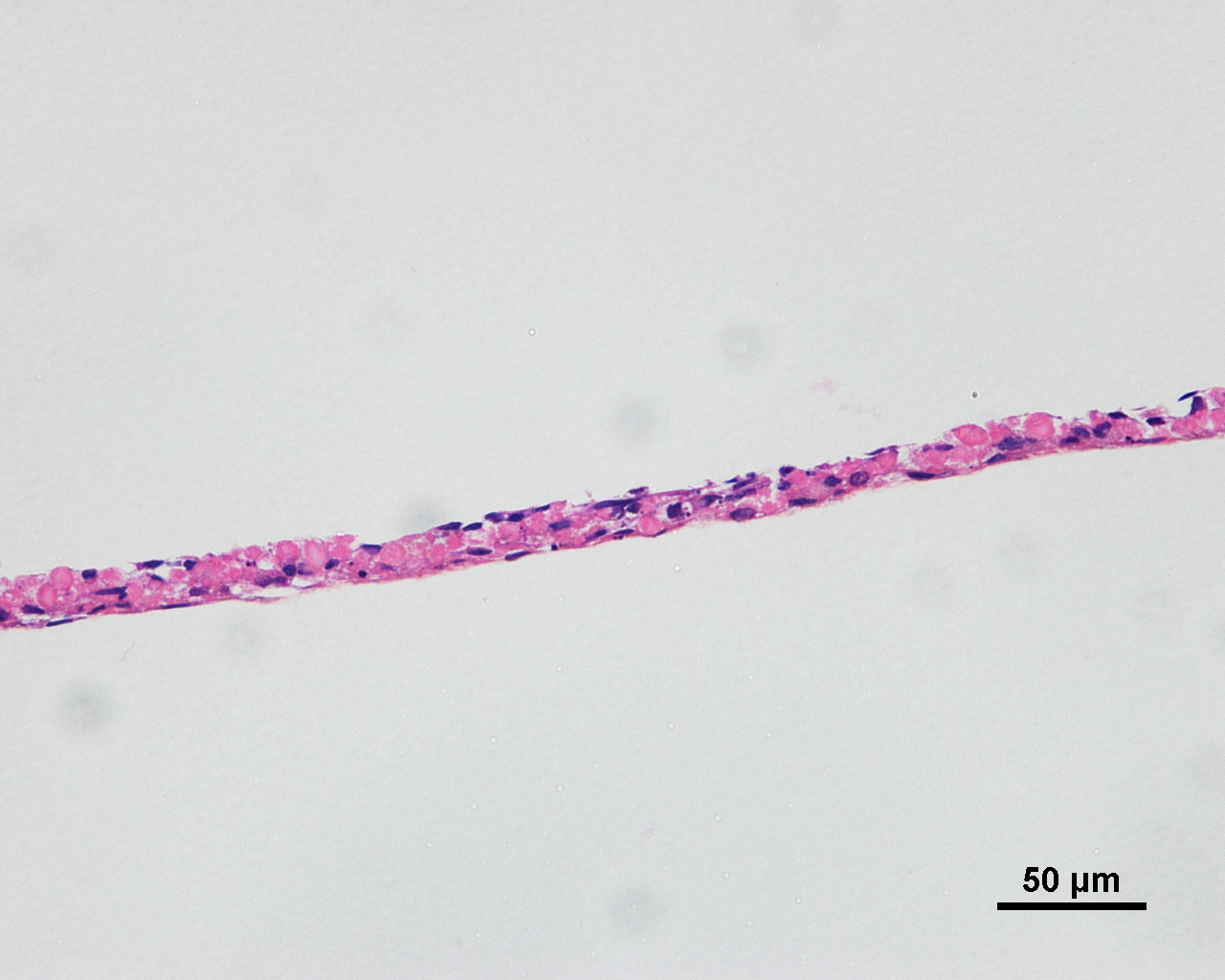

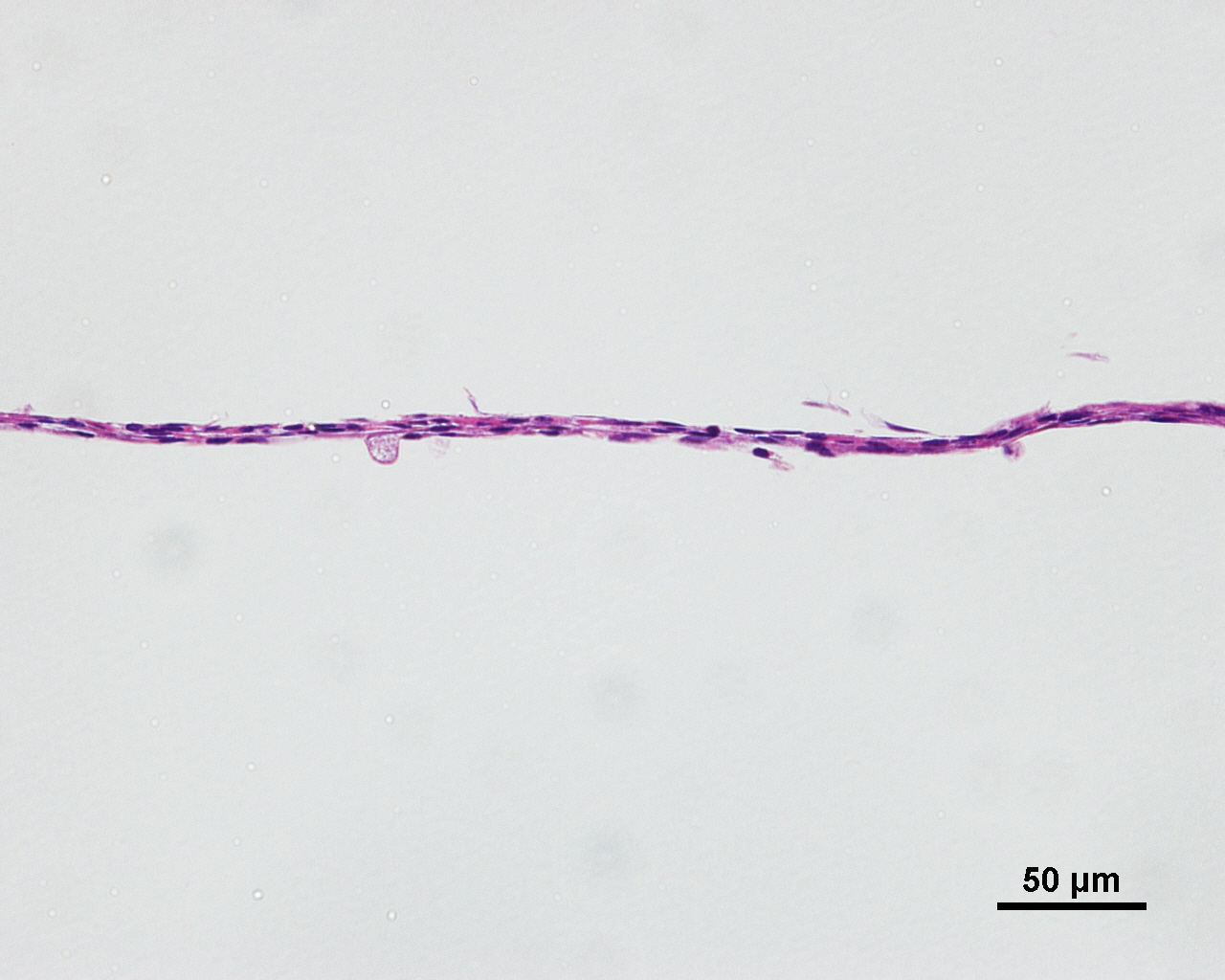

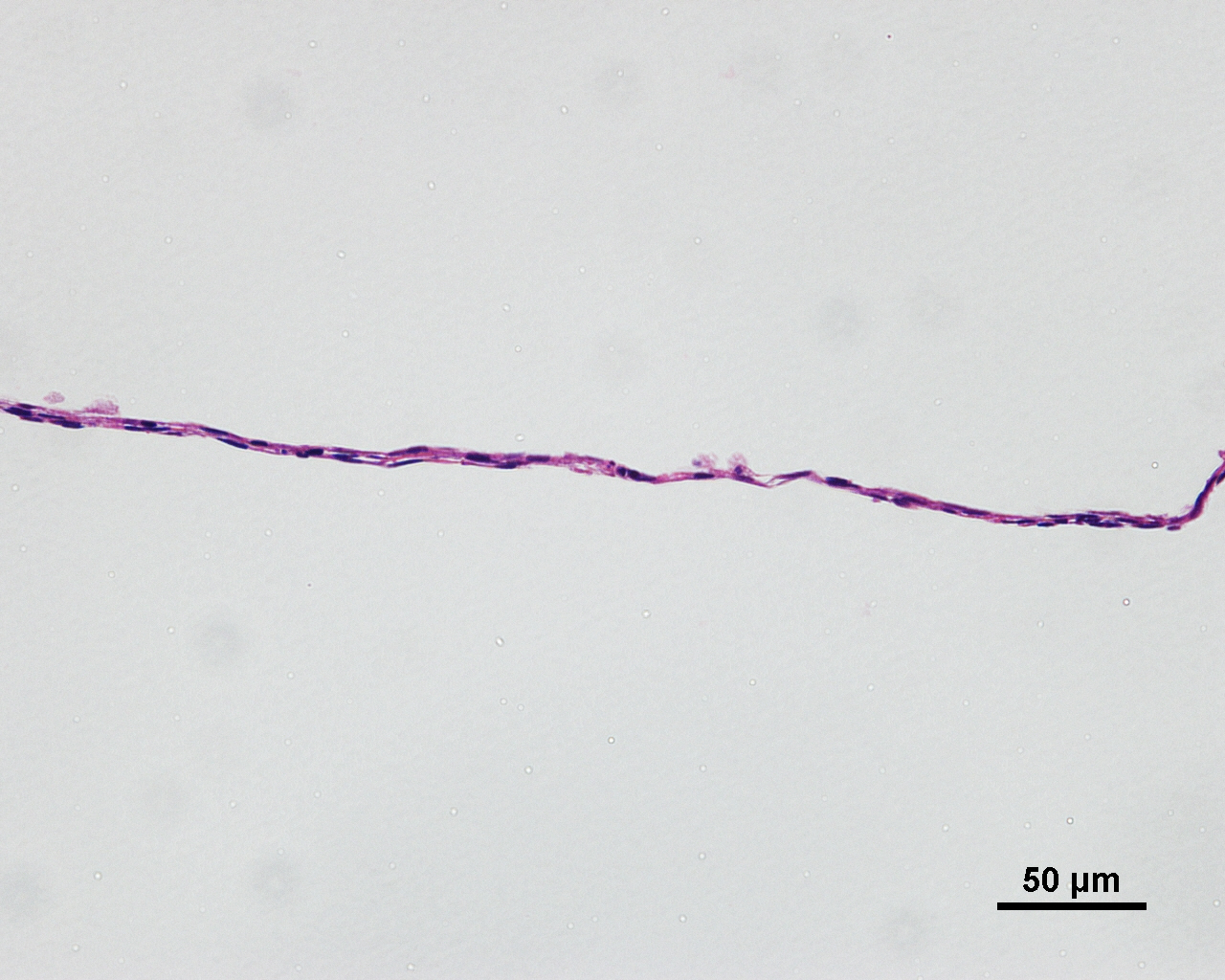

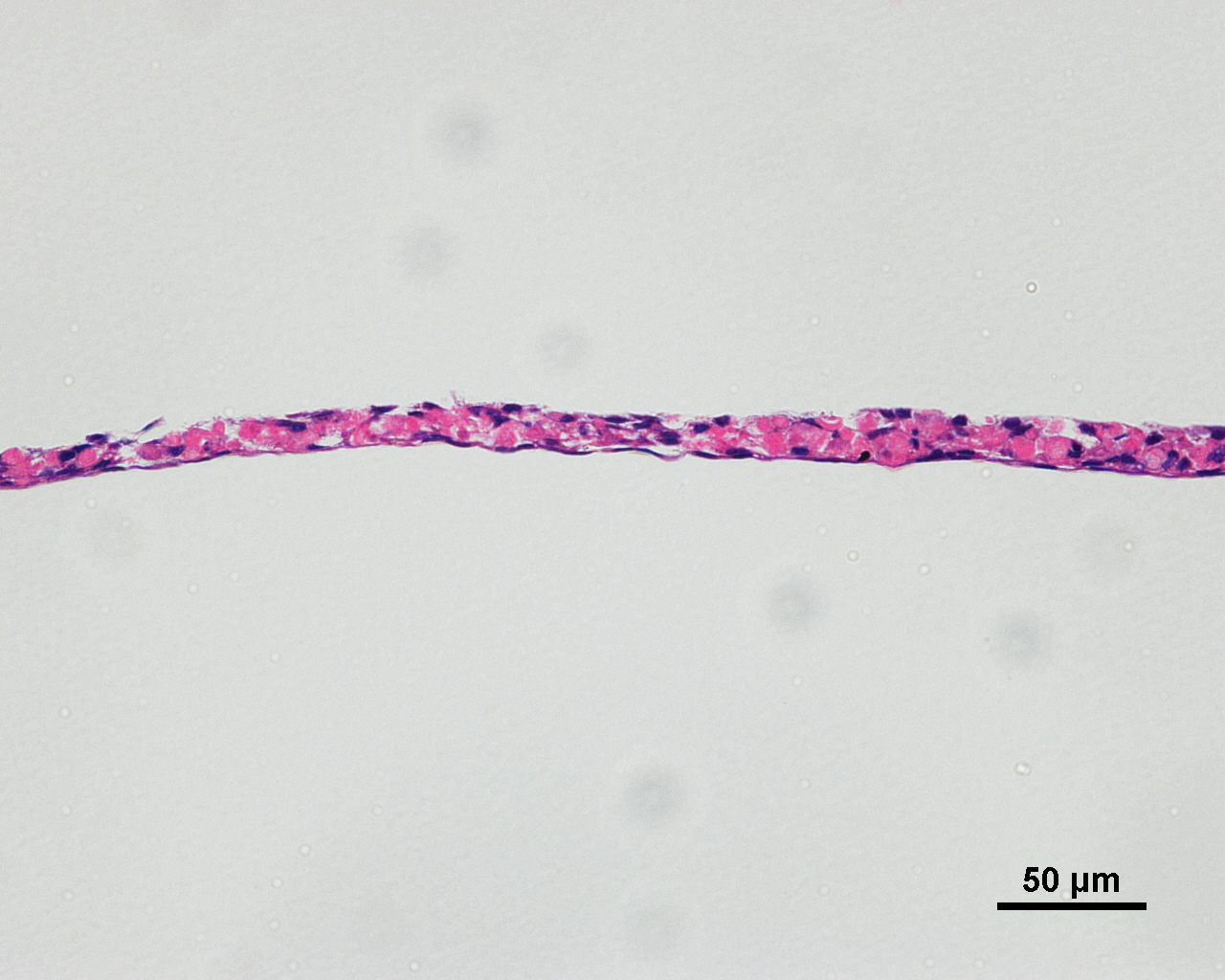


**C**

**D**

**A**

**B**

**E**

**F**

**19.5°C**

**23°C**

**26.5°C**

**30°C**

**33.5°C**

**37°C**

Figure S2. Hematoxylin–eosin-stained sections of single-layered cardiac cell sheets cultured for 3 days at various temperatures: (A) 19.5°C, (B) 23°C, (C) 26.5°C, (D) 30°C, (E) 33.5°C, and (F) 37°C. The thicknesses of cardiac cell sheets cultured between 19.5°C and 30°C were almost the same. However, cultivation at 33.5°C and 37°C produced thinner cardiac cell sheets (scale bar, 50 μm).


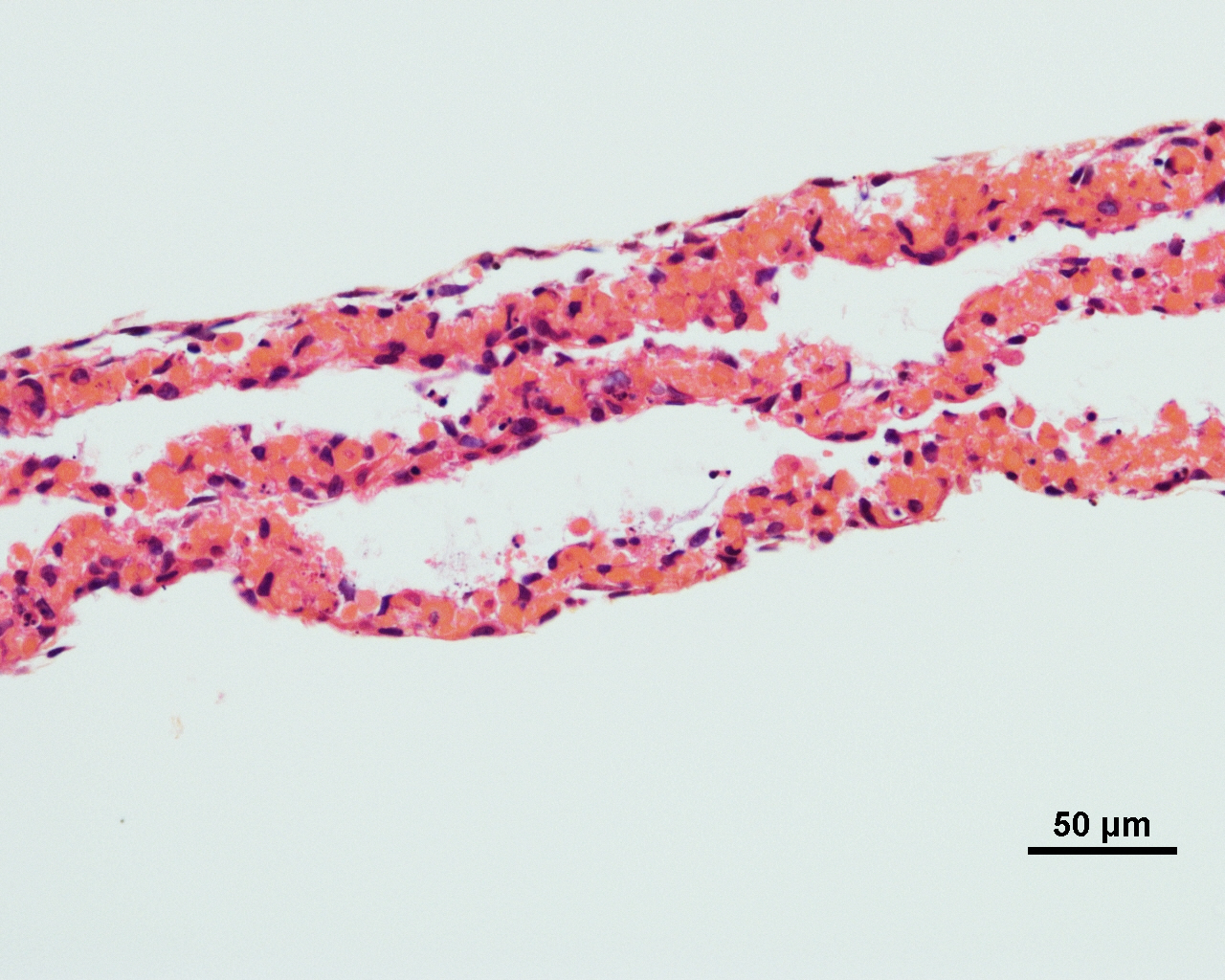

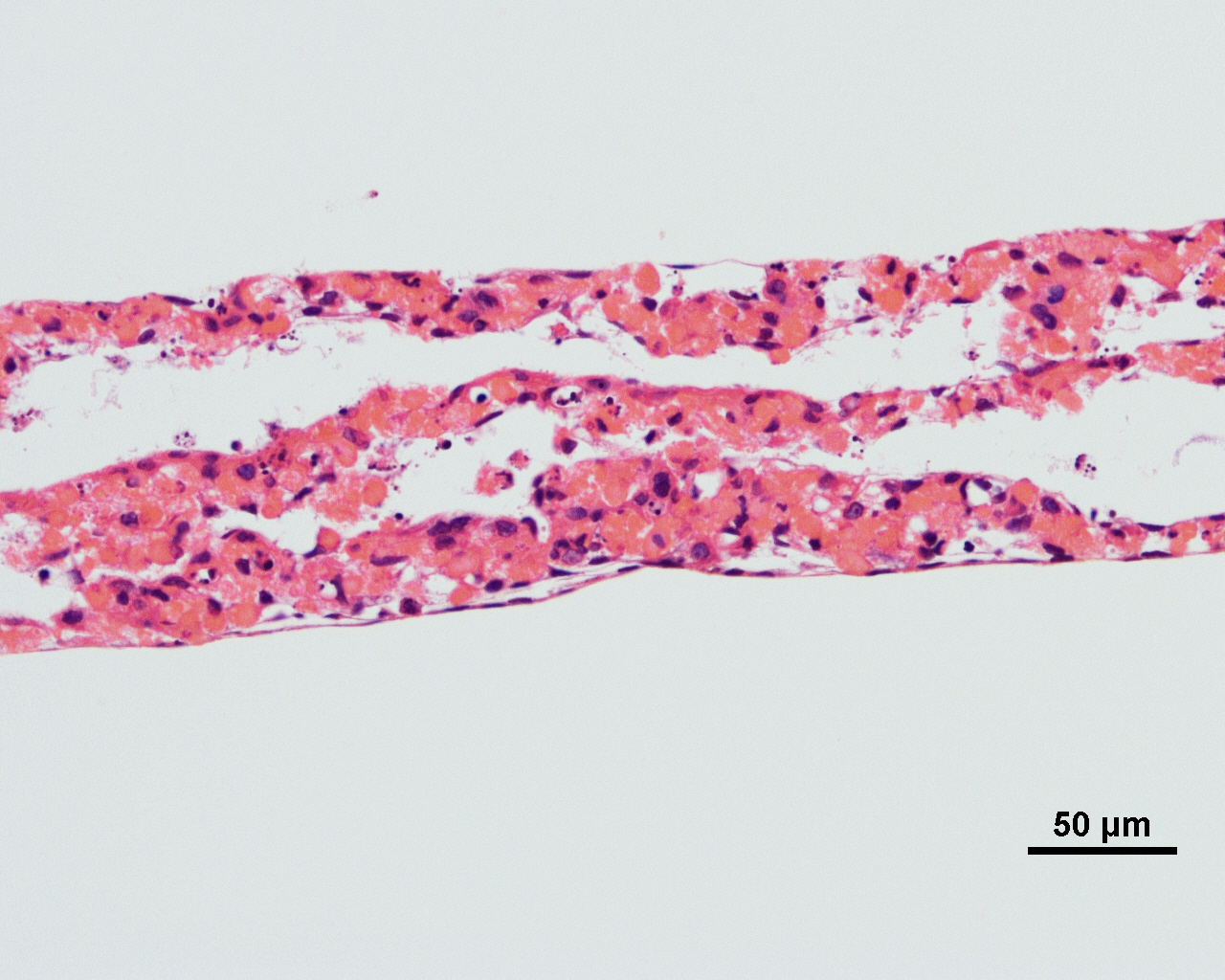

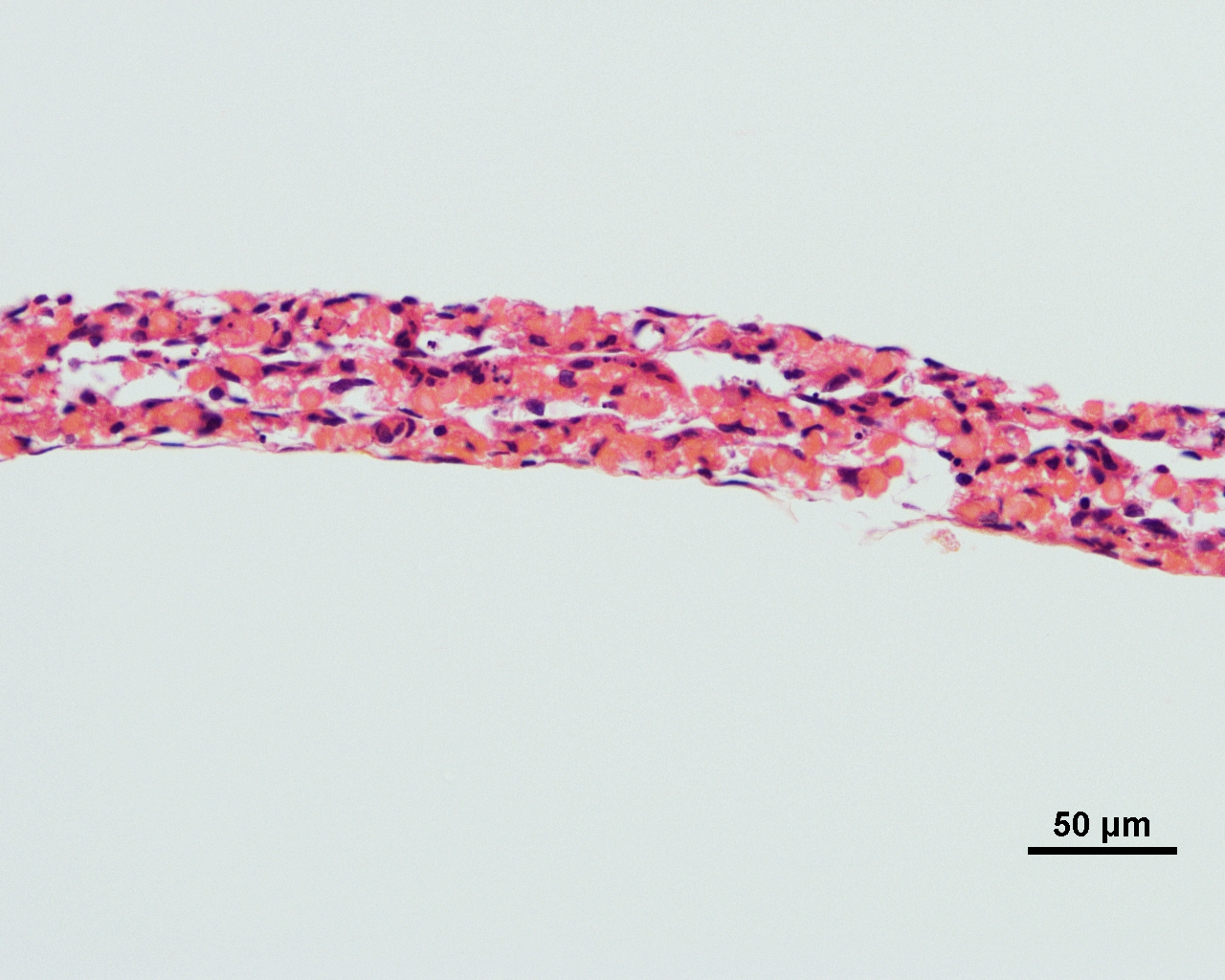

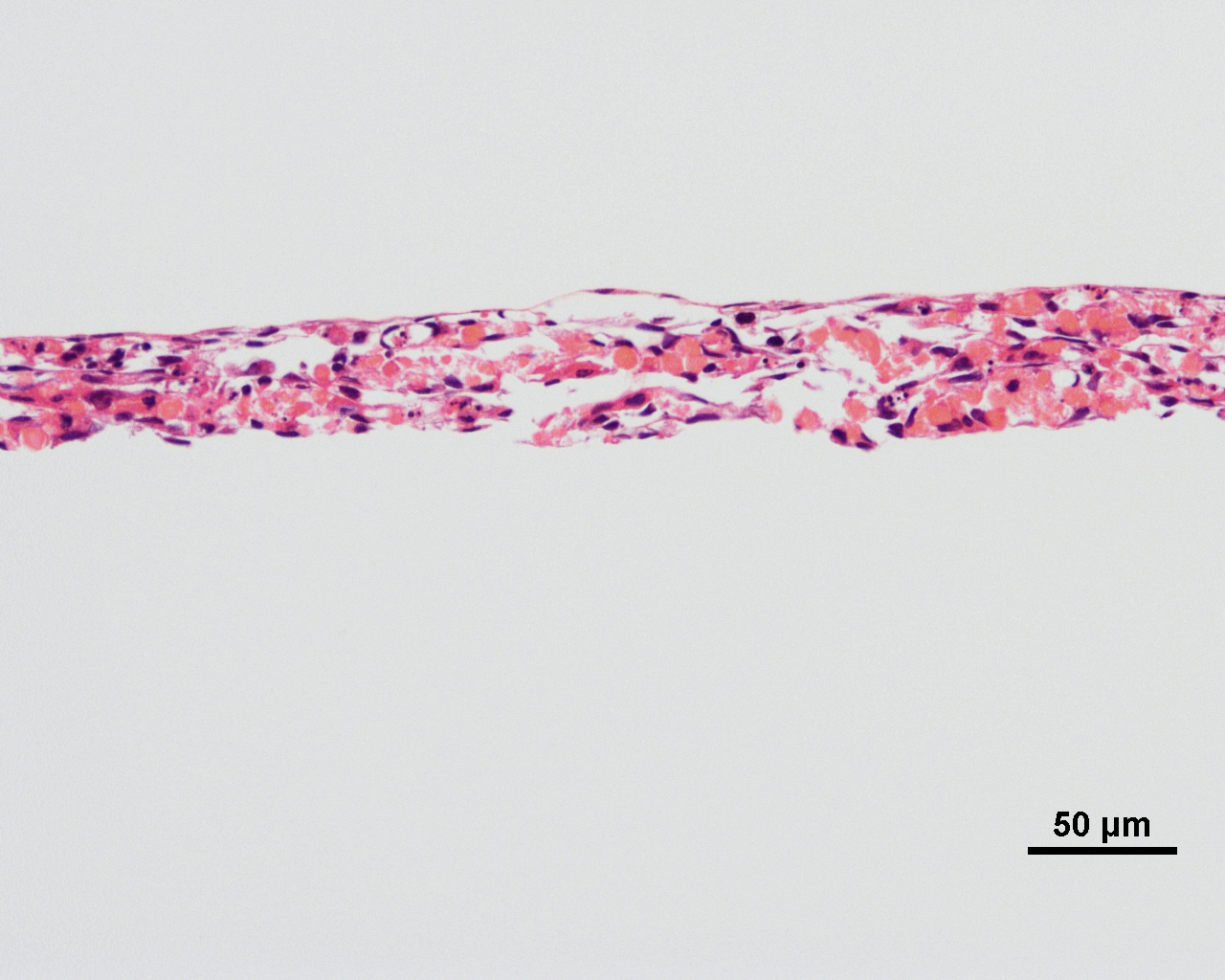

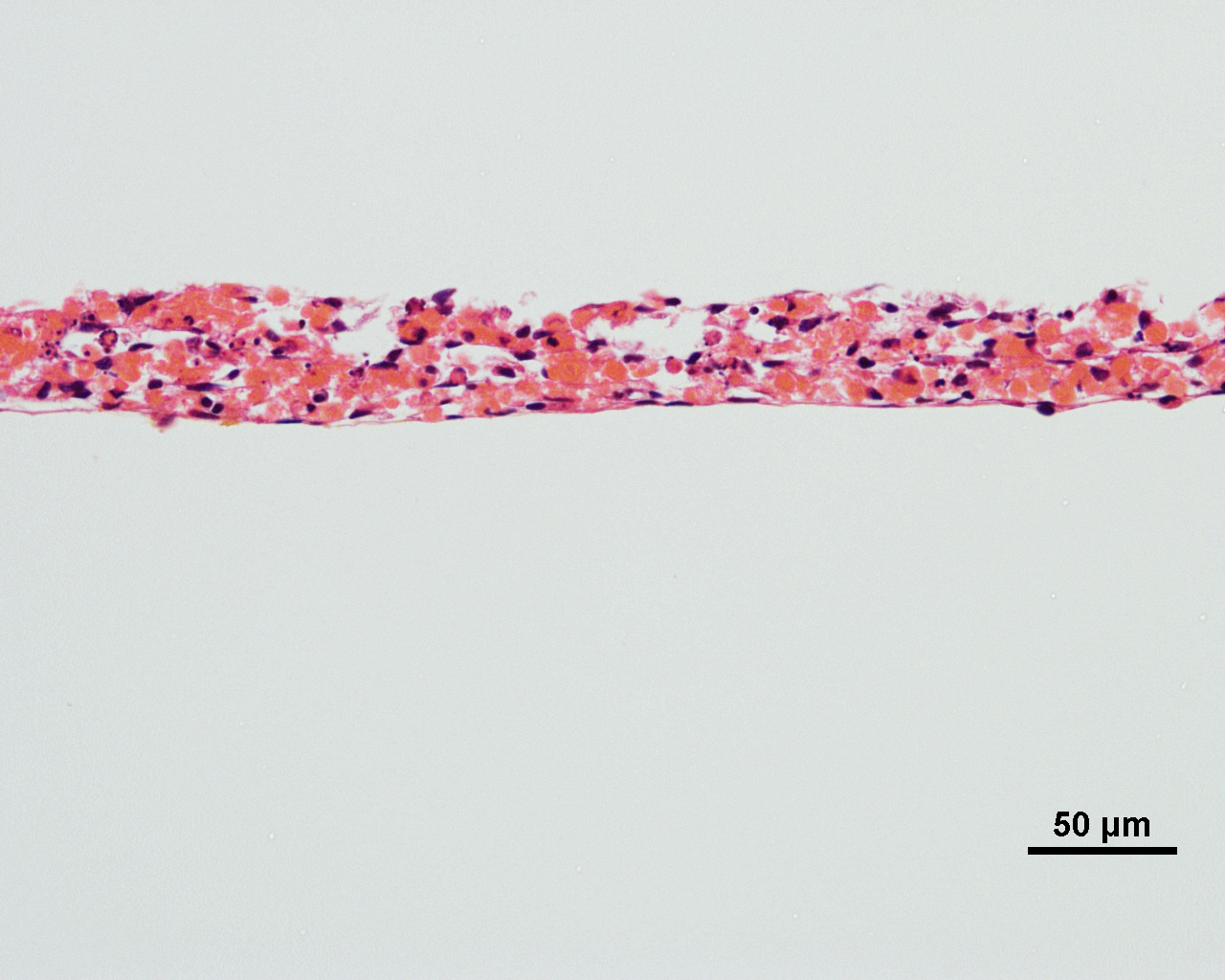

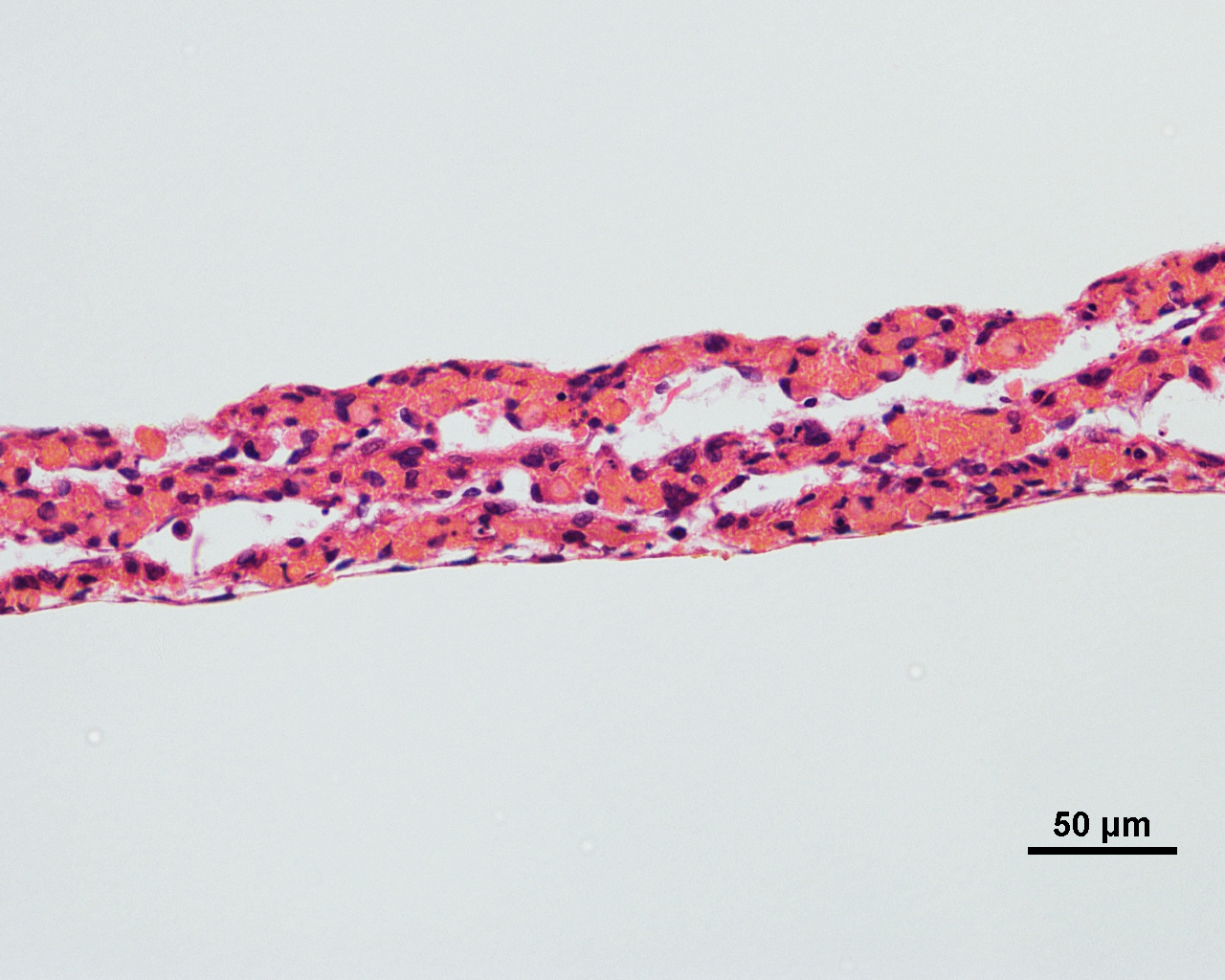


**C**

**D**

**A**

**B**

**E**

**F**

**19.5°C**

**23°C**

**26.5°C**

**30°C**

**33.5°C**

**37°C**

Figure S3. Hematoxylin–eosin-stained specimens of triple-layered cardiac cell sheets were cultured for 3 days at various temperatures: (A) 19.5°C, (B) 23°C, (C) 26.5°C, (D) 30°C, (E) 33.5°C, and (F) 37°C. The gaps among cell layers were observed upon cultivation between 19.5°C and 26.5°C (scale bar, 50 μm).


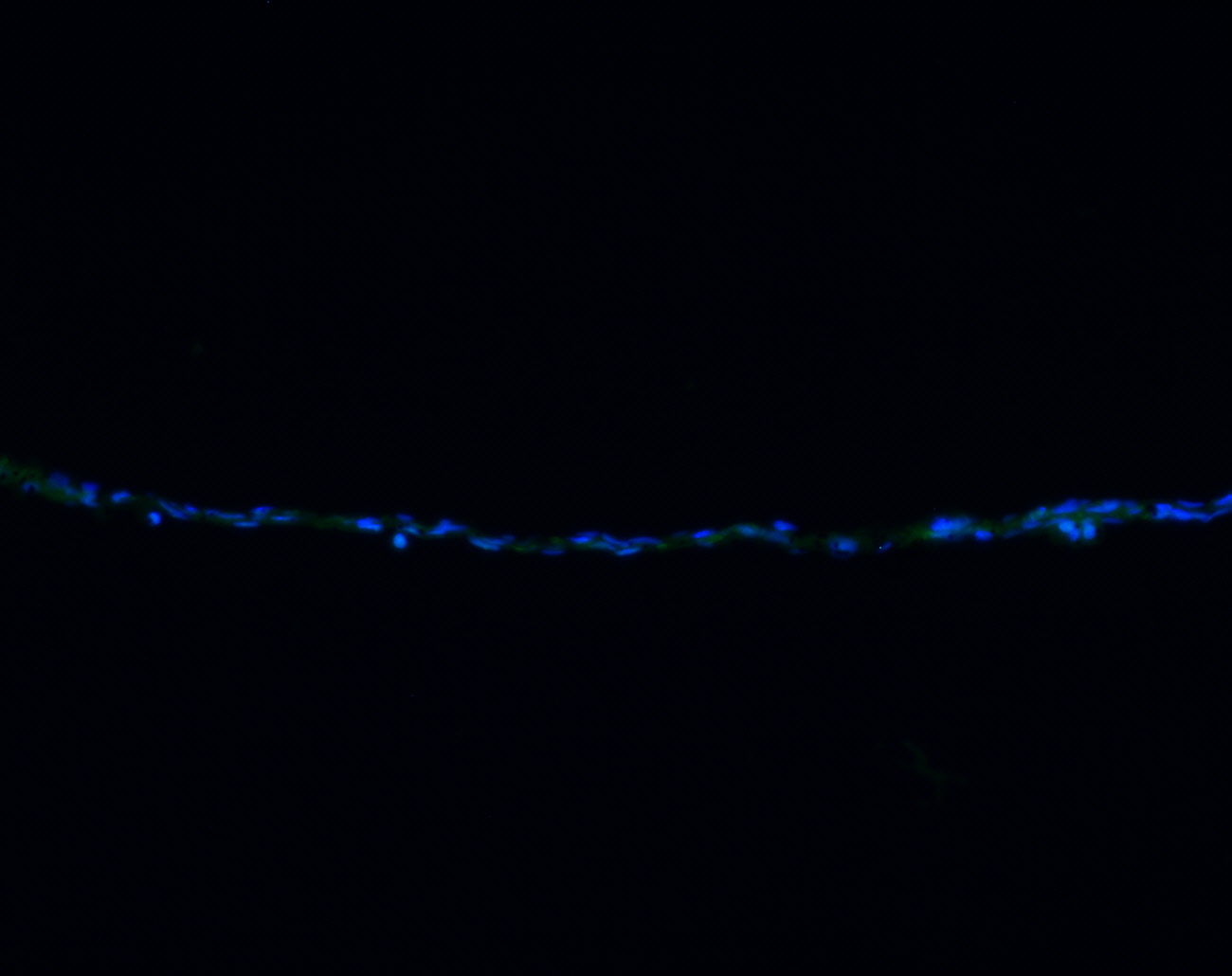

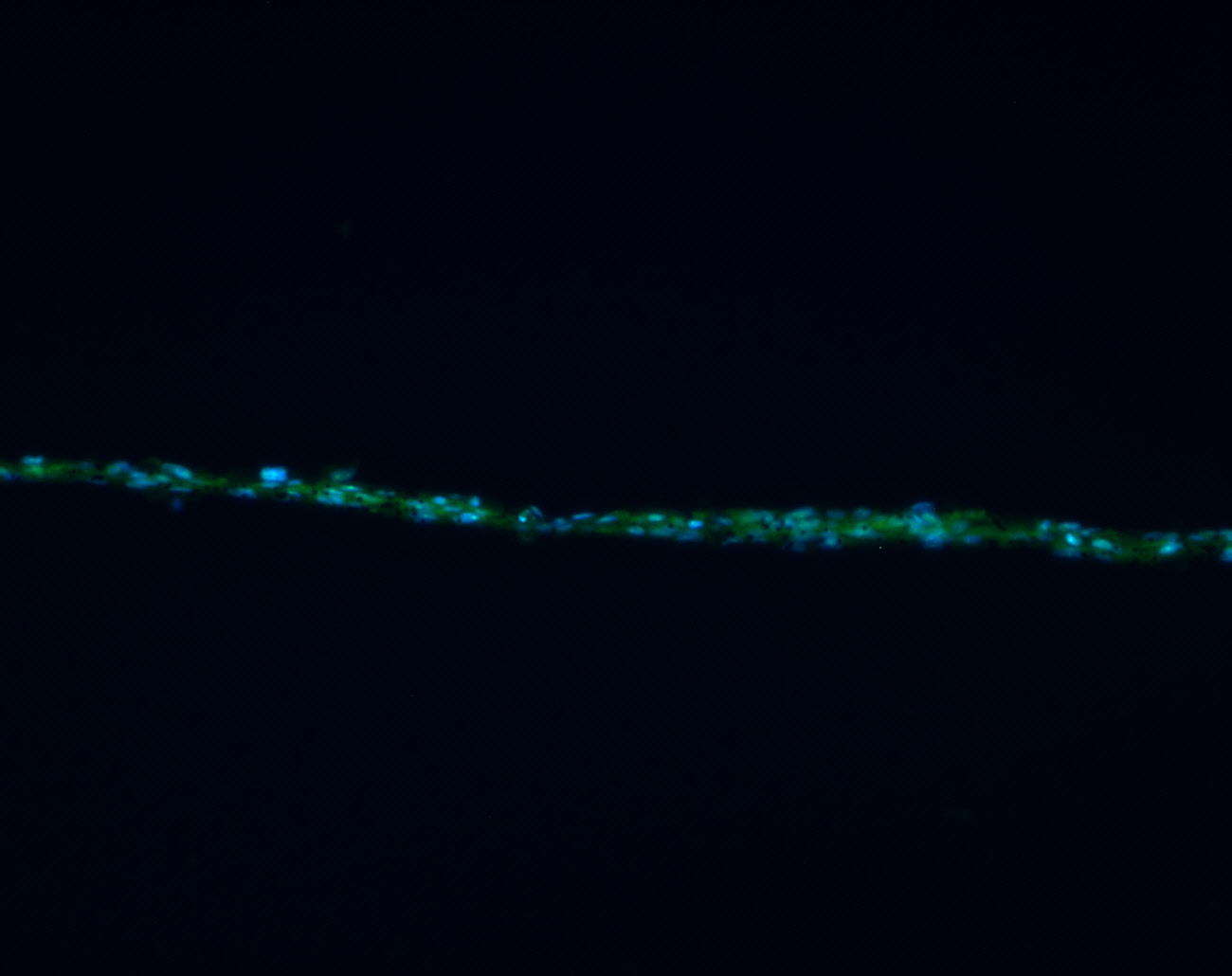

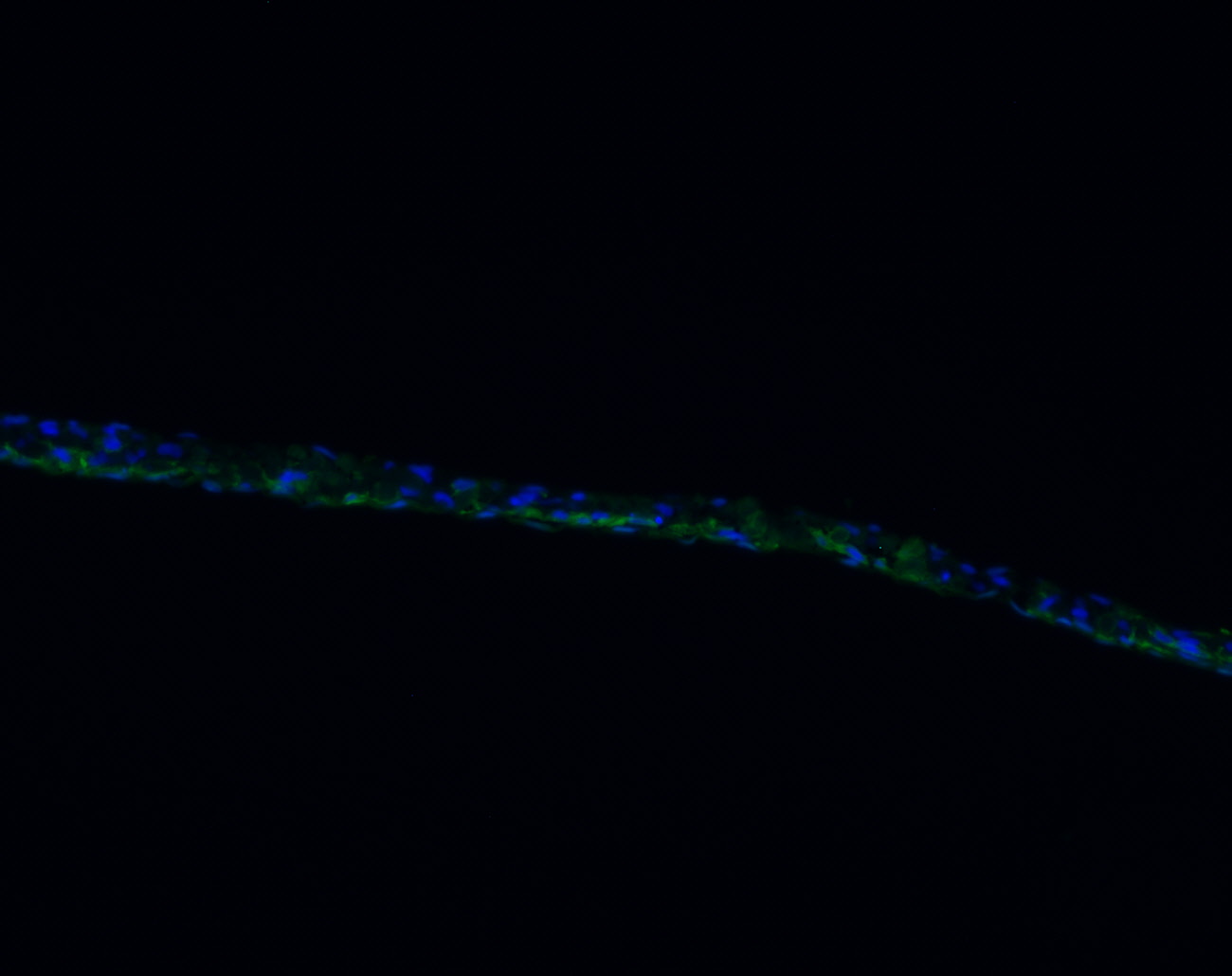

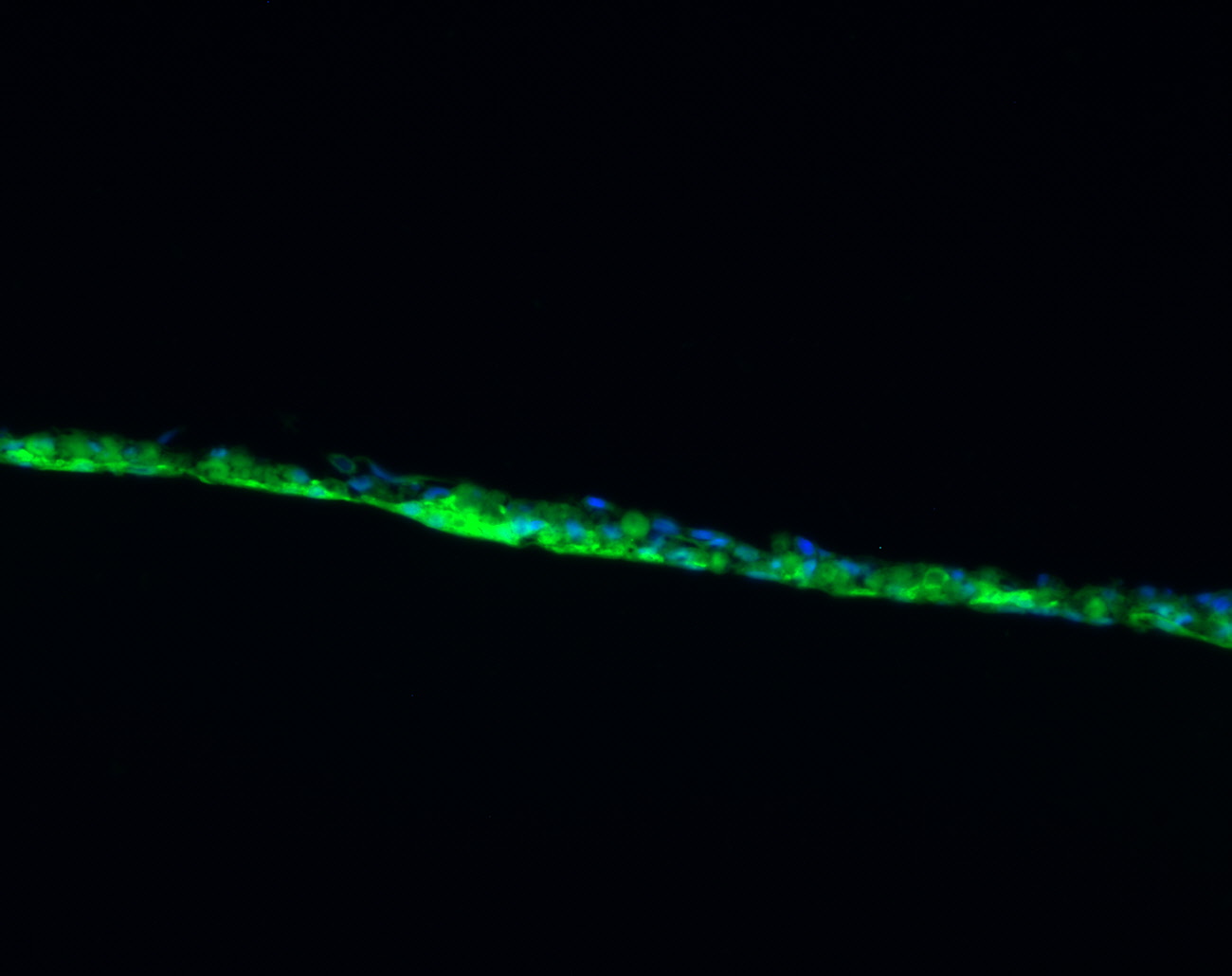

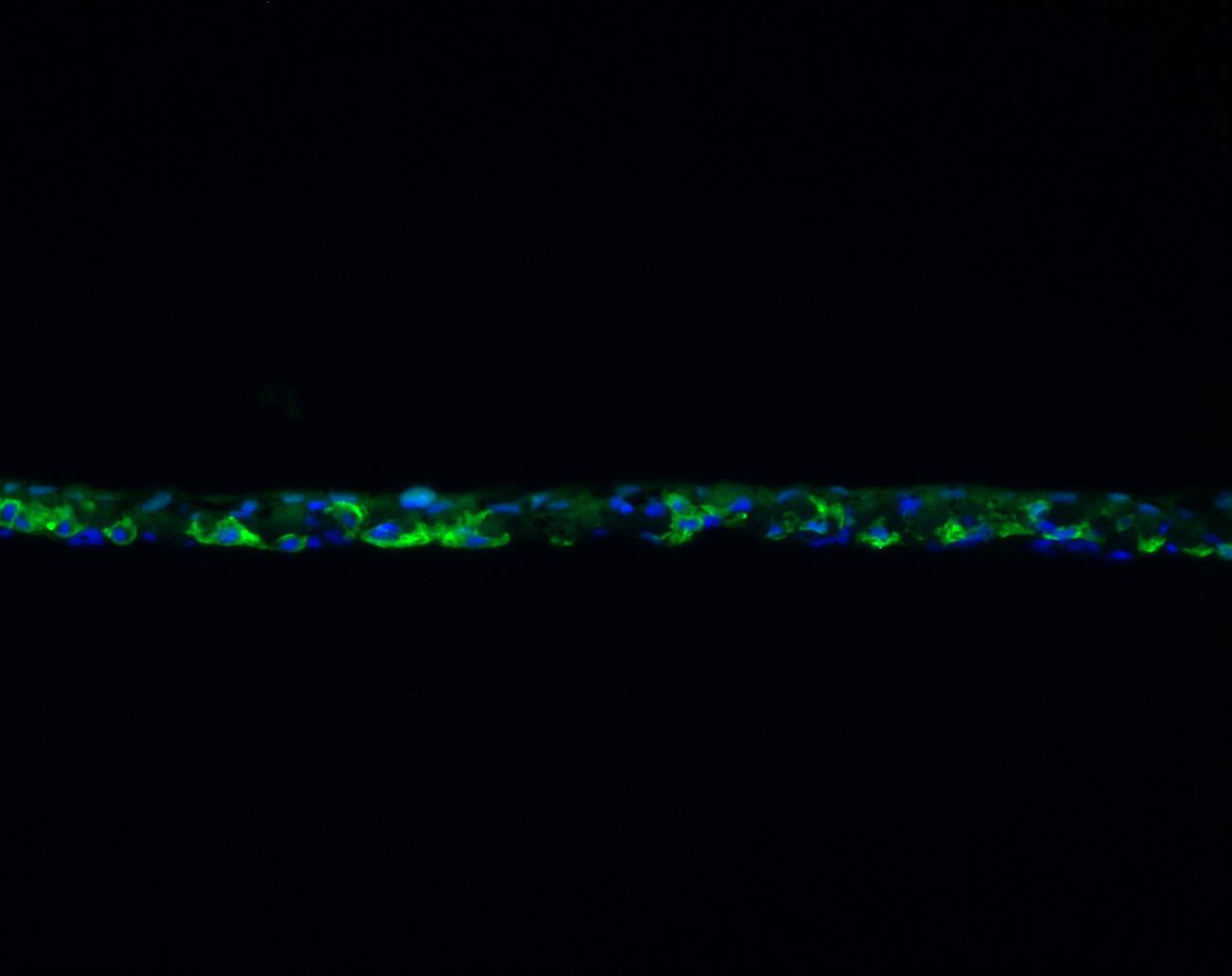

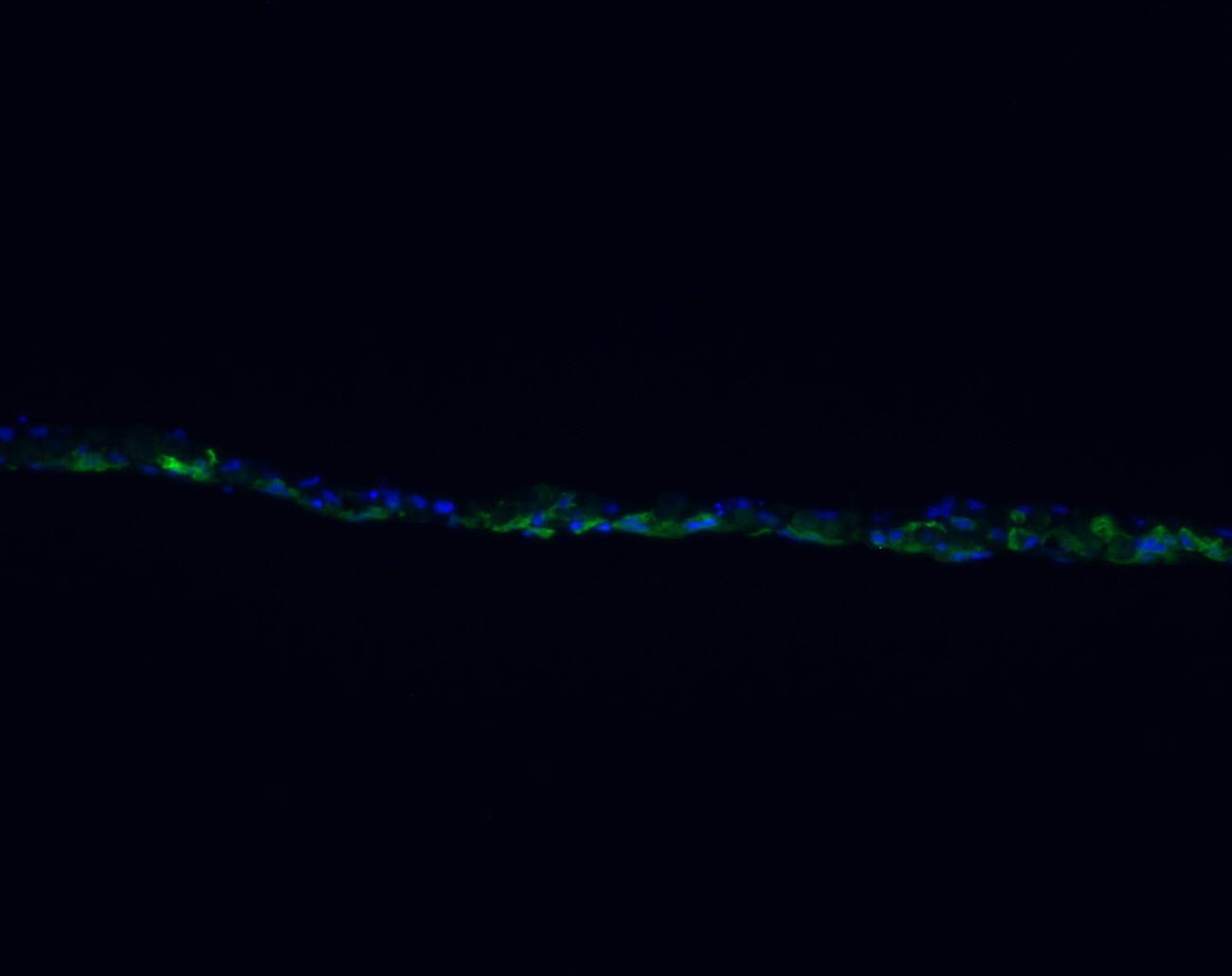


C

A

B

F

D

26.5°C

19.5°C

23°C

37°C

30°C

E

33.5°C

Figure S4. Troponin-stained sections of single-layered cardiac cell sheets cultured for 3 days at various temperatures: (A) 19.5°C, (B) 23°C, (C) 26.5°C, (D) 30°C, (E) 33.5°C, and (F) 37°C. Green shows troponin T and blue shows cell nuclei. No green markers were found in cell sheets cultured at 37°C (F) (scale bar, 50 μm).


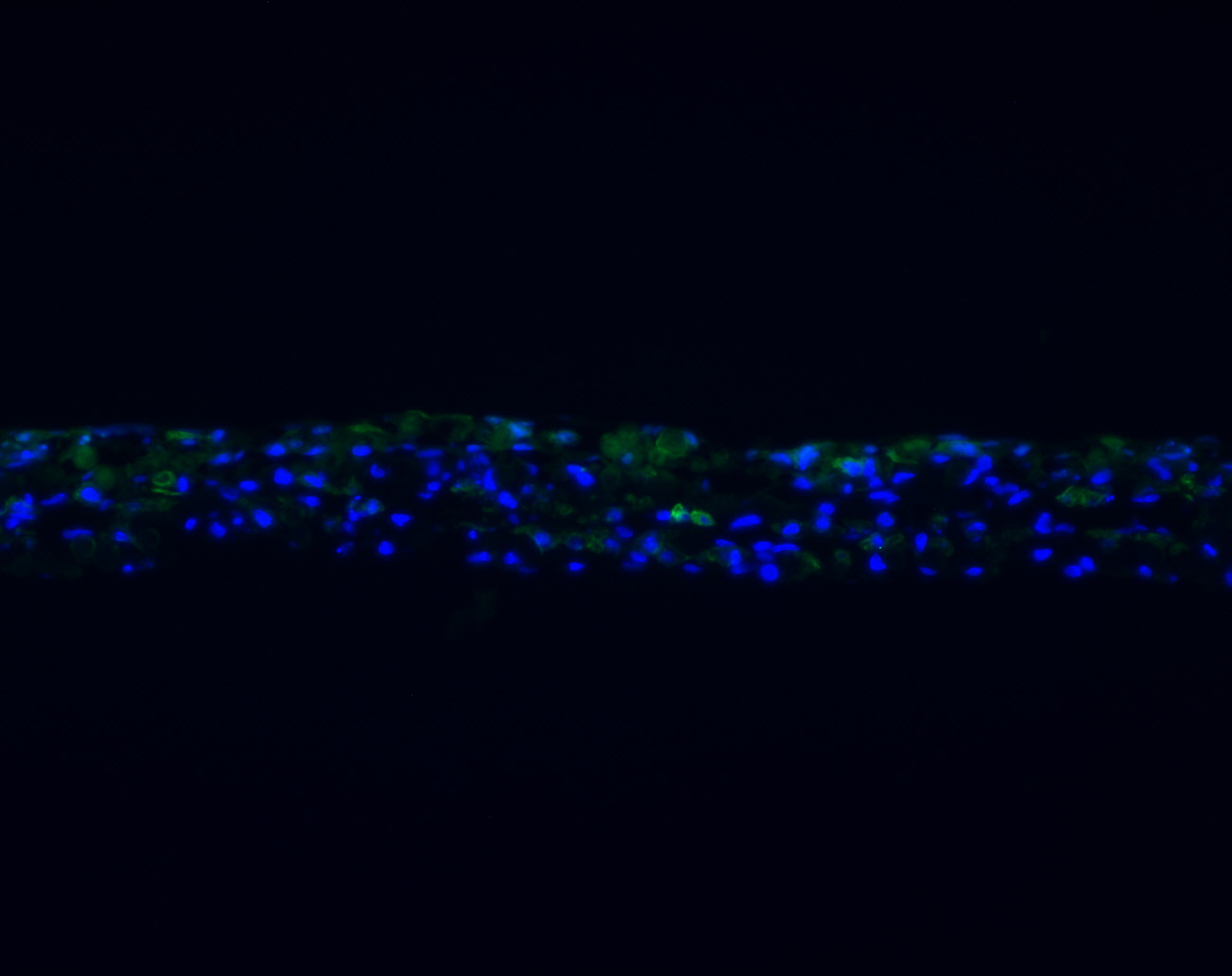

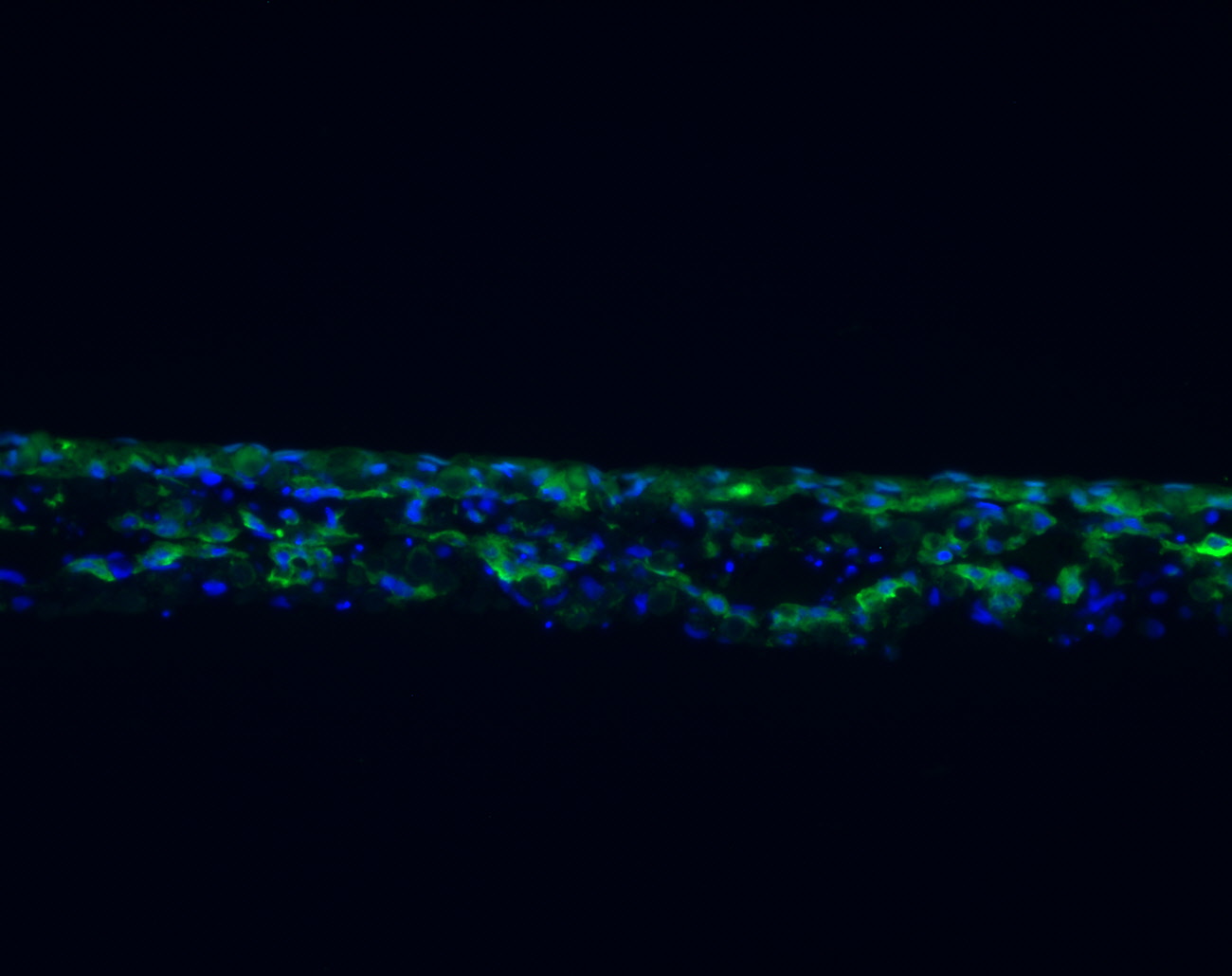

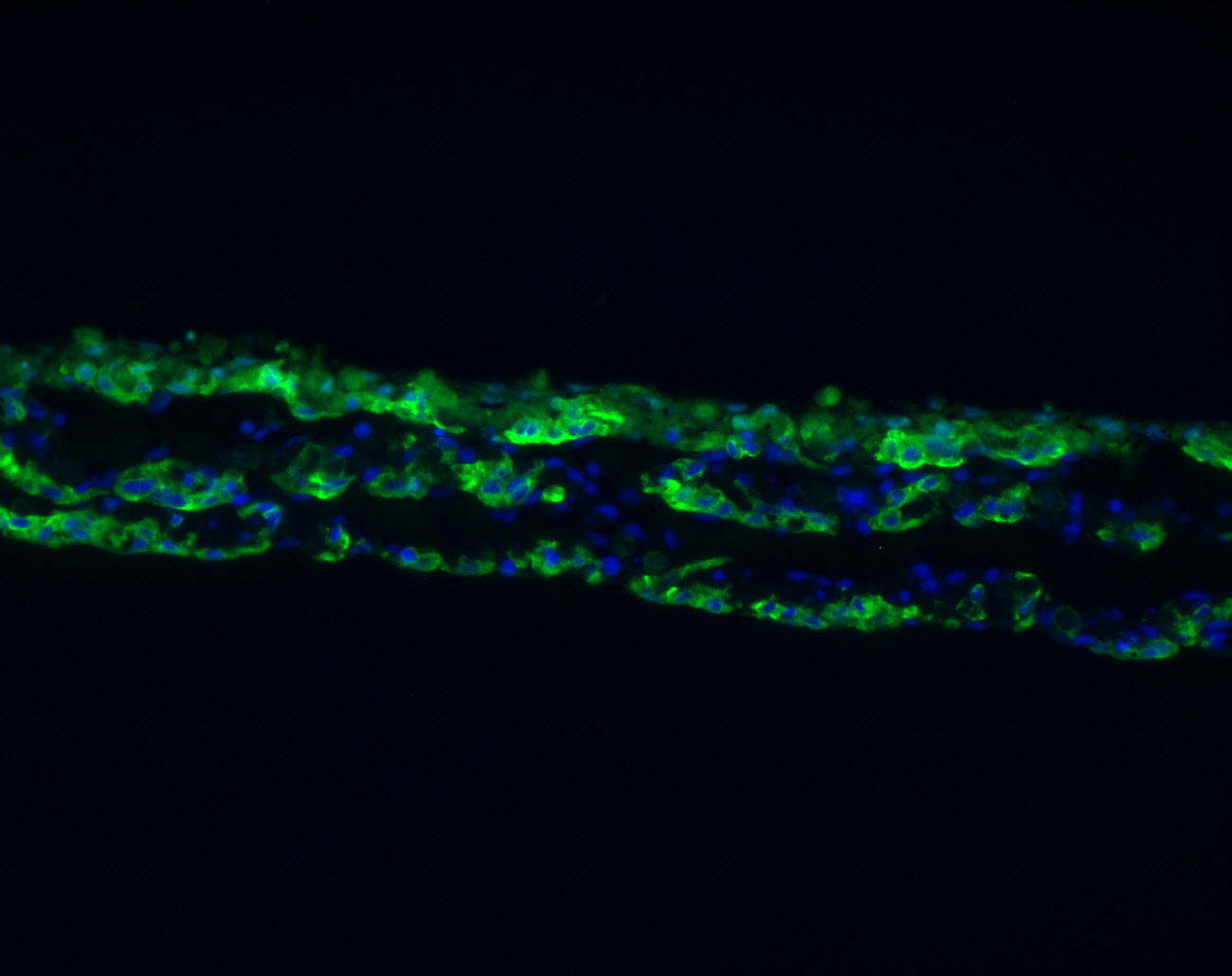

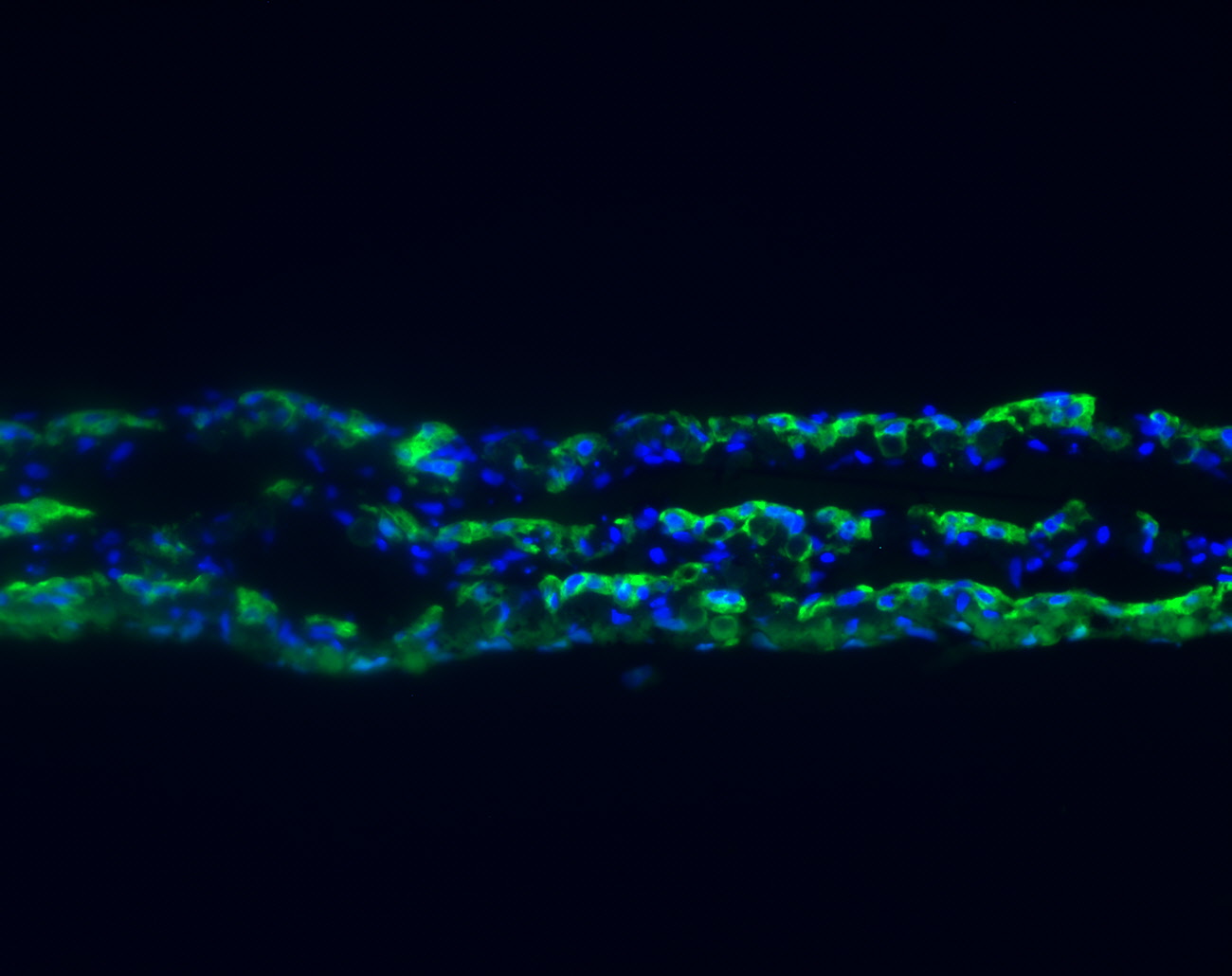

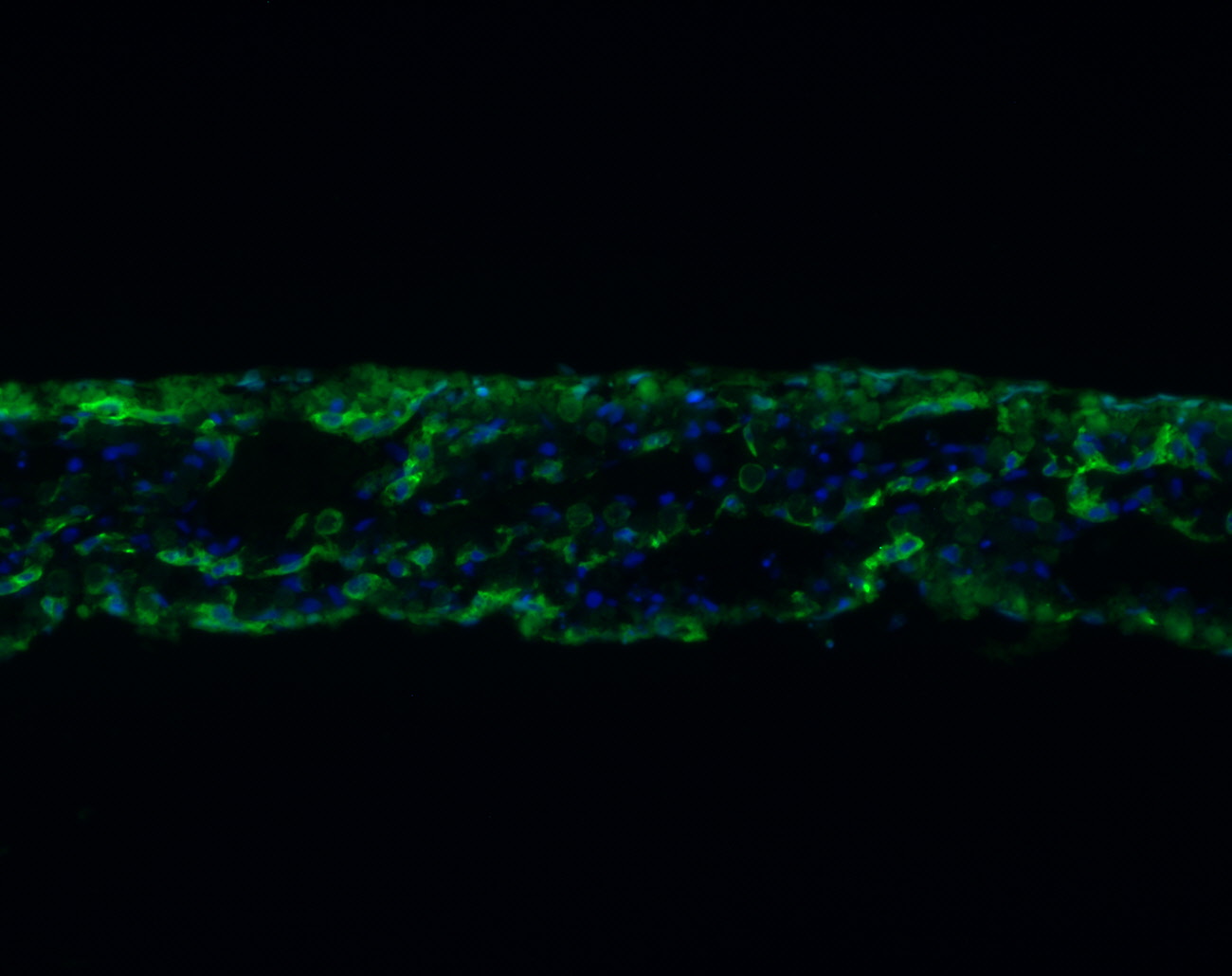

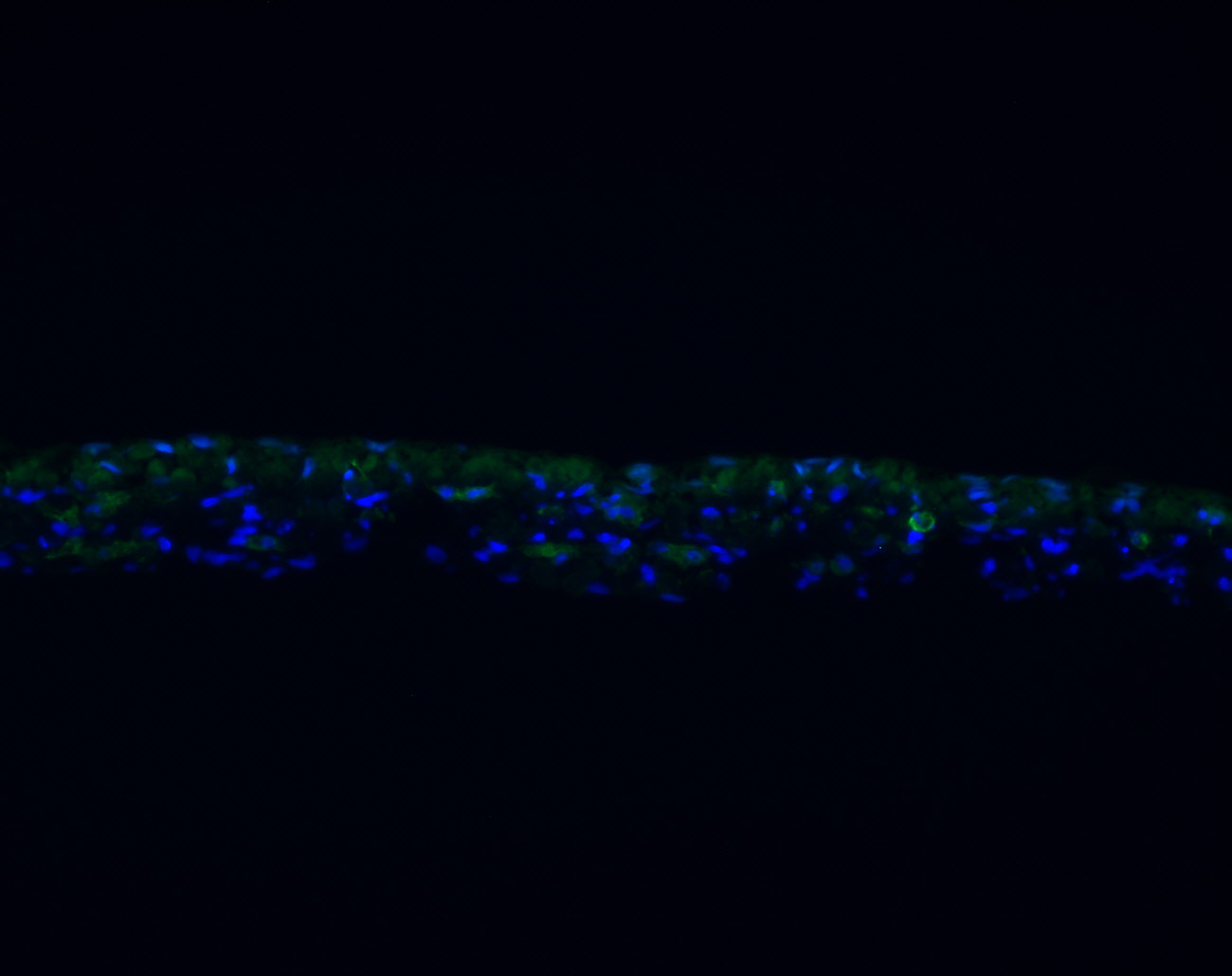


C

D

A

B

E

F

19.5°C

23°C

26.5°C

30°C

33.5°C

37°C

Figure S5. Troponin-stained sections of triple-layered cardiac cell sheets cultured for 3 days at various temperatures: (A) 19.5°C, (B) 23°C, (C) 26.5°C, (D) 30°C, (E) 33.5°C, and (F) 37°C. The green and blue areas in the microphotographs show troponin T and nuclei, respectively. No green area was observed in cell sheets cultured at 33.5°C or 37°C (E, F) (scale bar, 50 μm).


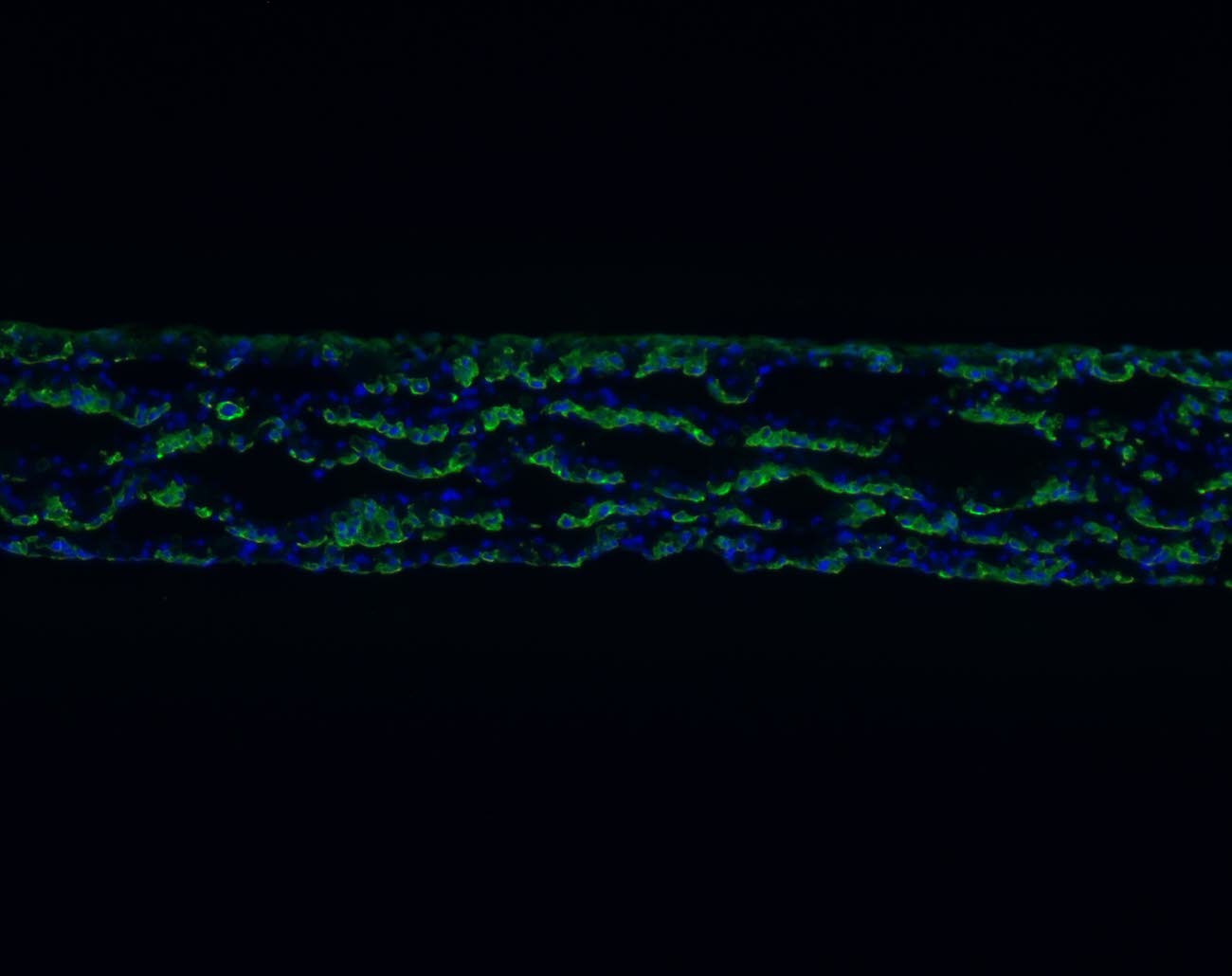

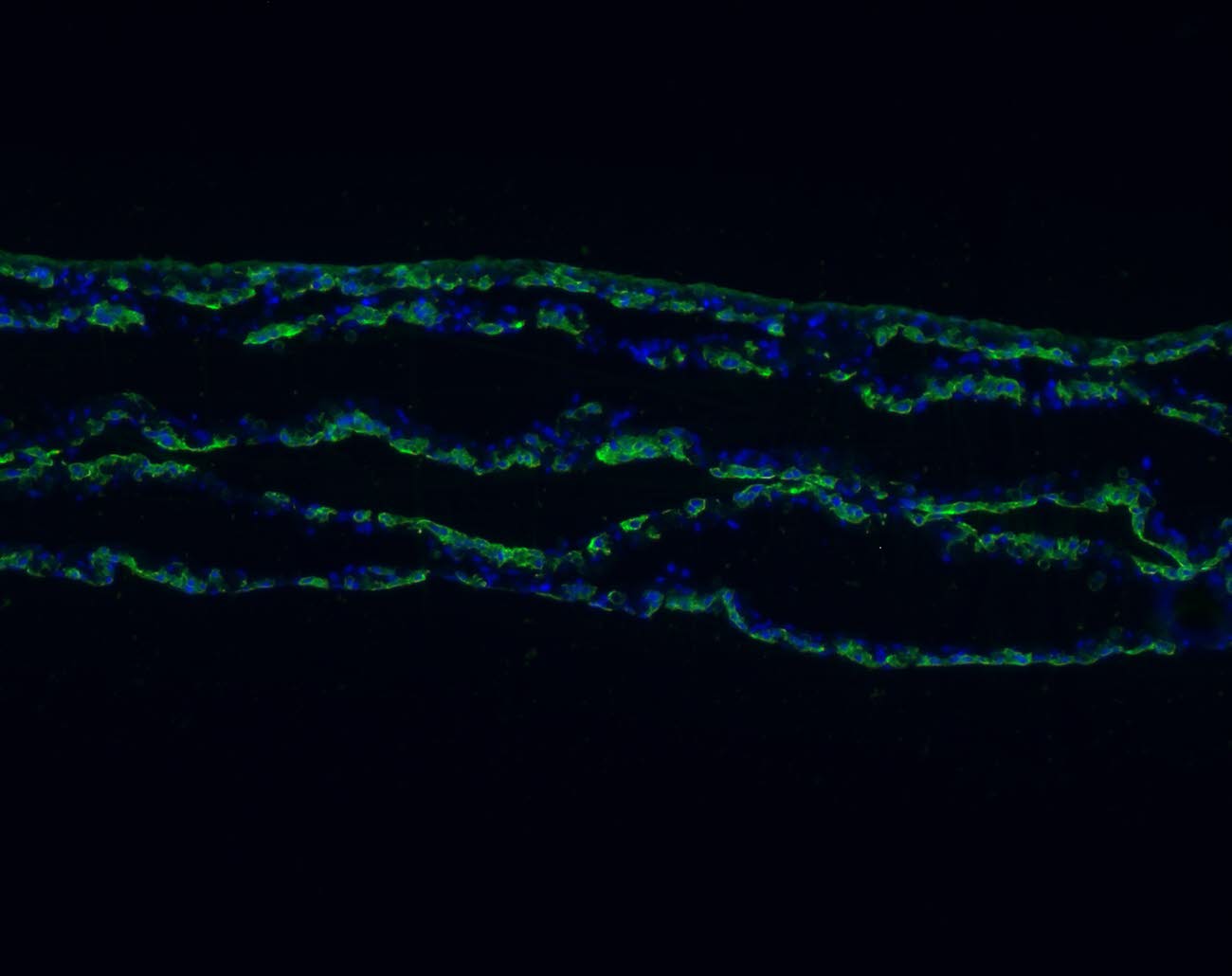

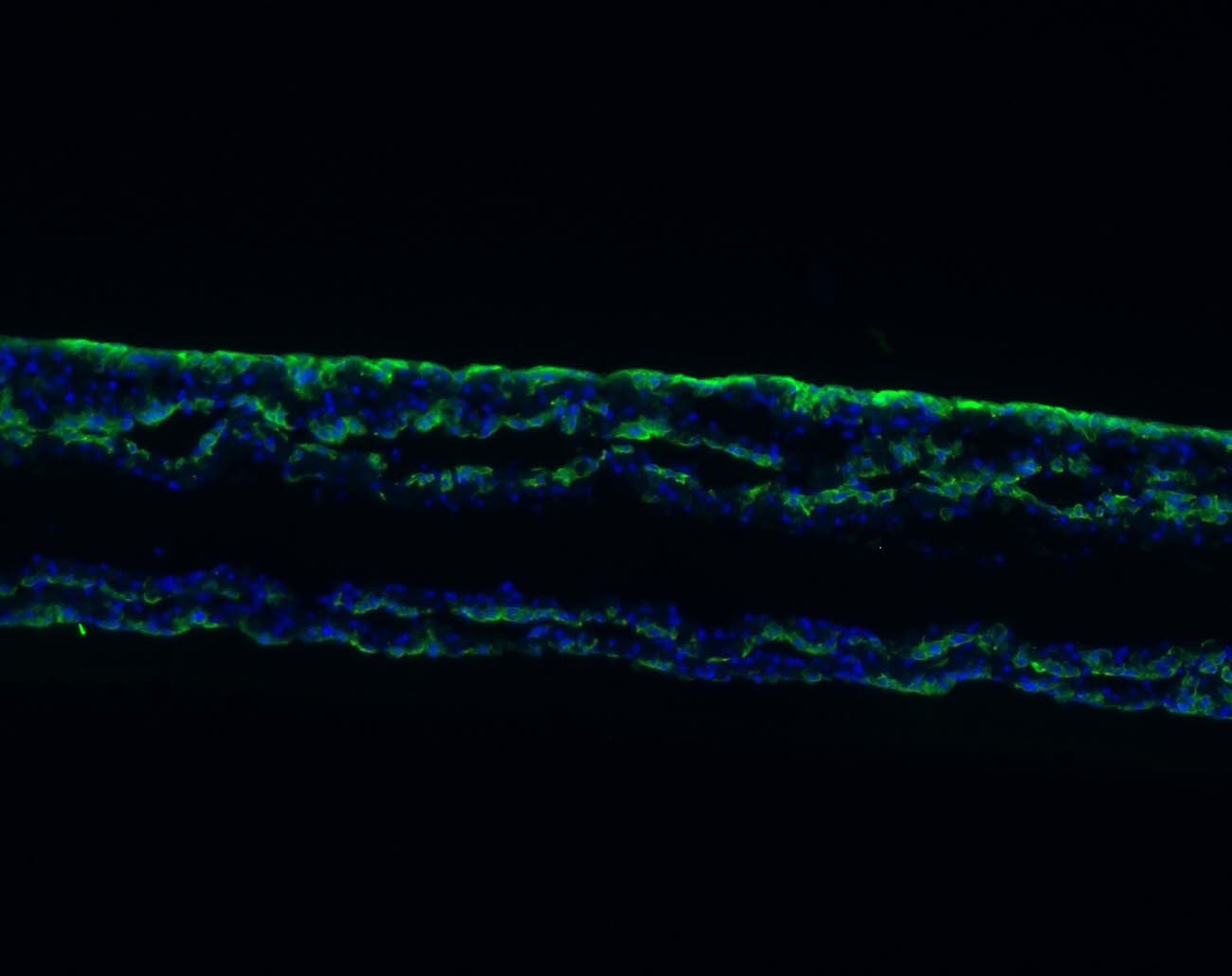

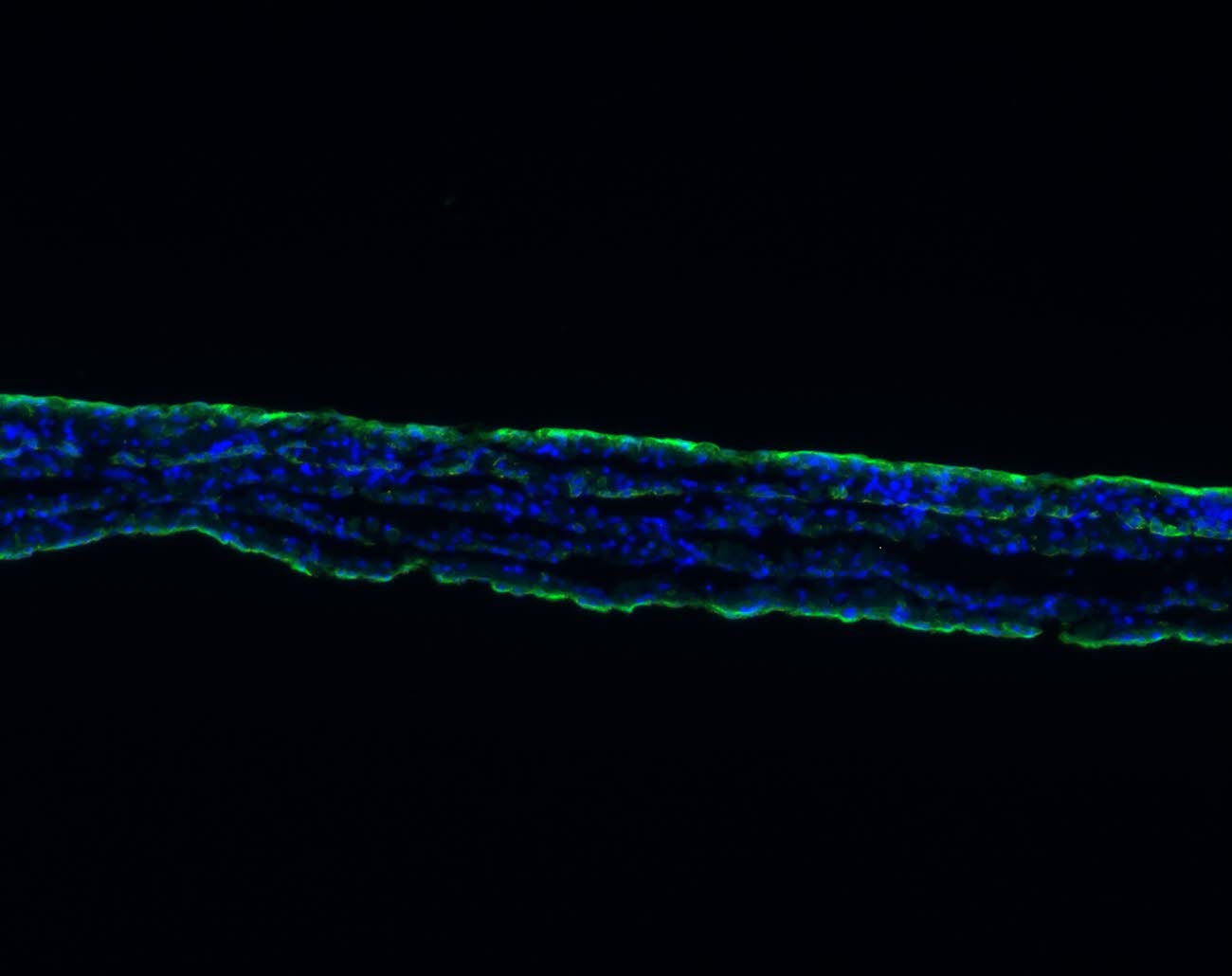

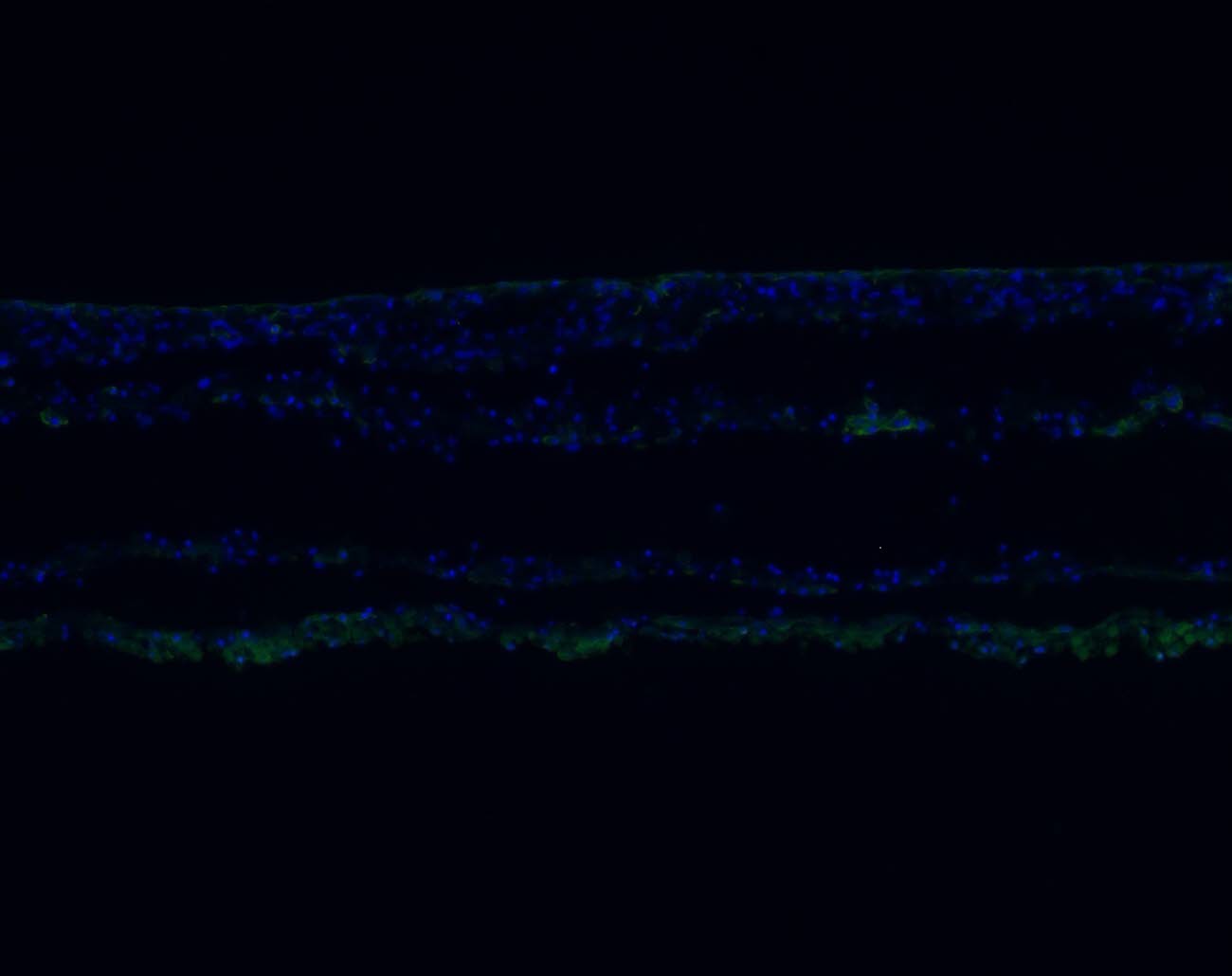

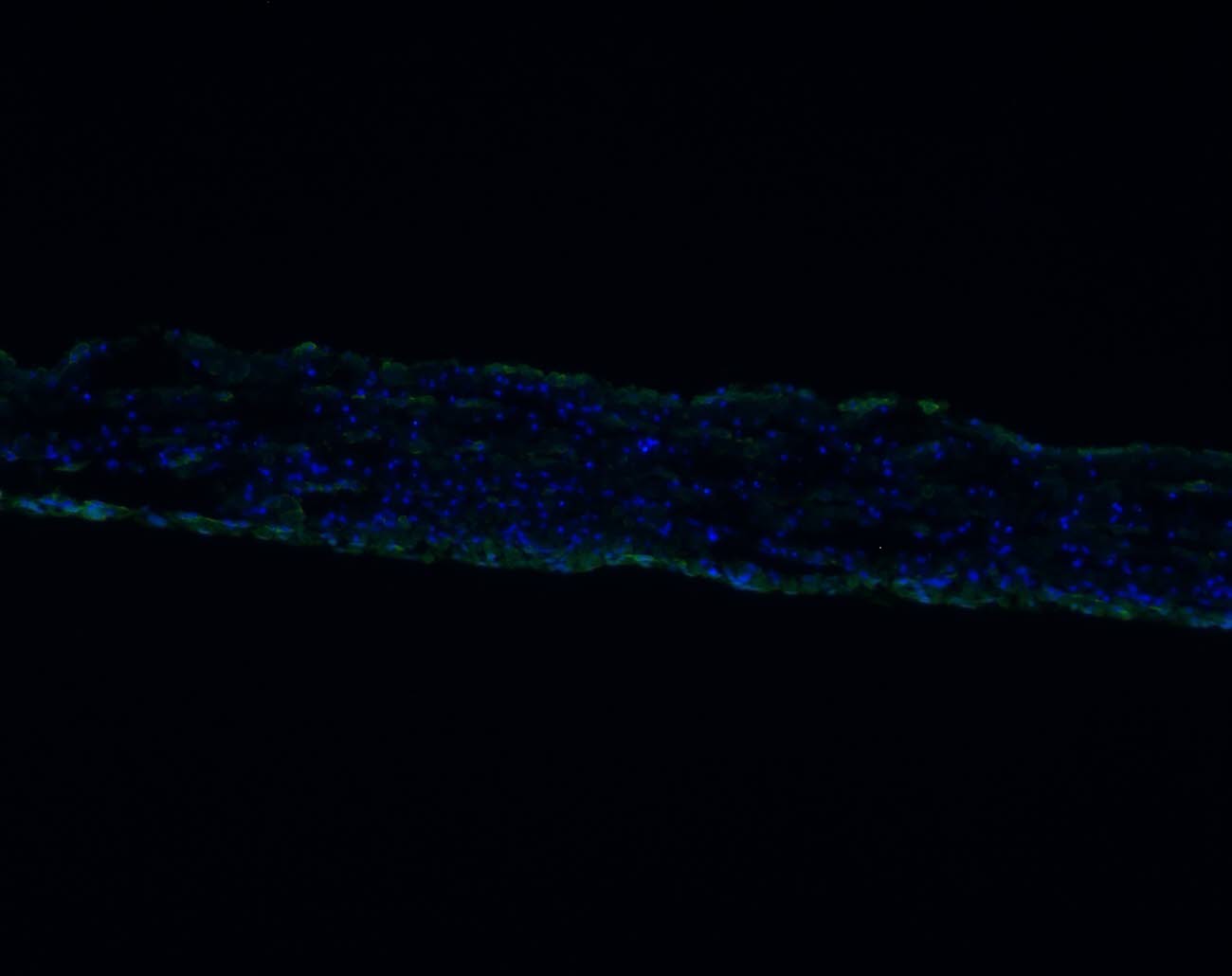


C

D

A

B

E

F

19.5°C

23°C

26.5°C

30°C

33.5°C

37°C

Figure S6. Troponin-stained sections of quintuple-layered cardiac cell sheets cultured for 3 days at various temperatures: (A) 19.5°C, (B) 23°C, (C) 26.5°C, (D) 30°C, (E) 33.5°C, and (F) 37°C. The green and blue areas in the microphotographs show troponin T and nuclei, respectively. No green area was observed in cell sheets cultured at 33.5°C or 37°C, and the results were largely similar to those of the triple-layered cell sheet (E, F) (scale bar, 50 μm).


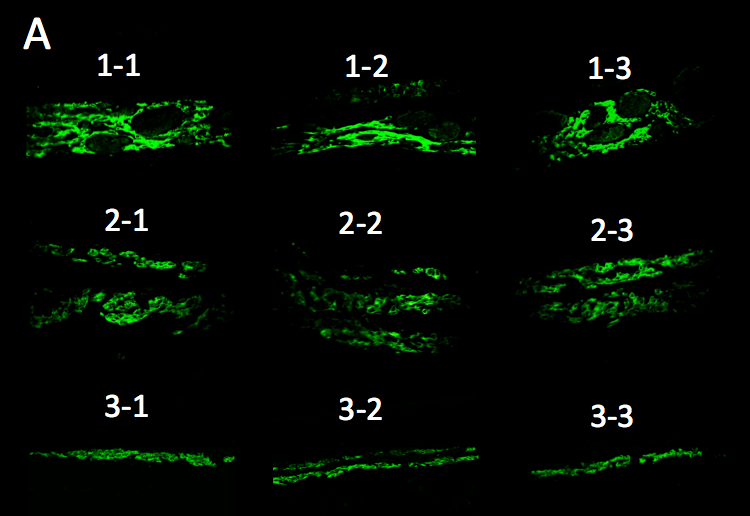

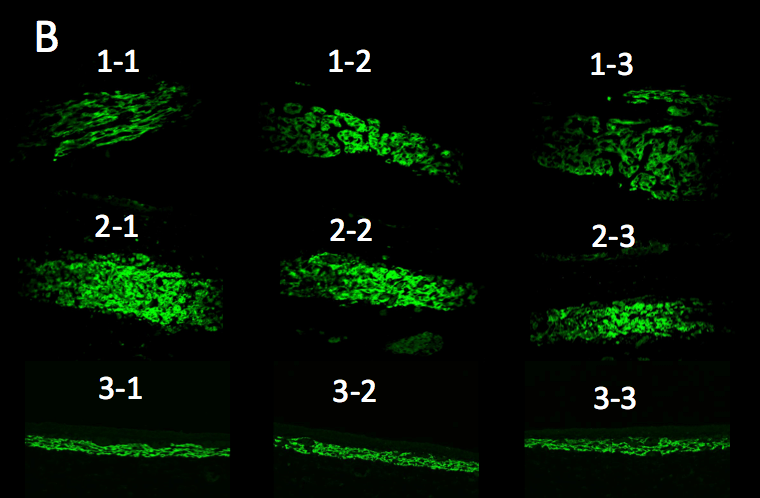


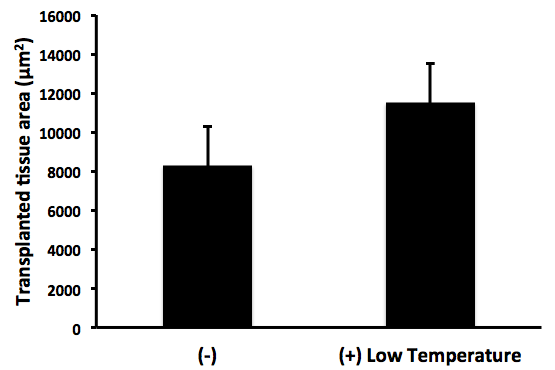


**C**

Figure S7. Troponin-stained sections of quintuple-layered cardiac cell sheets transplanted for 2 weeks. (A) Images are sections of transplanted cardiac cell sheets after 2 weeks without the low-temperature treatment. No. 1-1, 1-2, and 1-3 represent the same tissue and were obtained from three different points. No. 2-1, 2-2, and 2-3 and 3-1, 3-2, and 3-3 also each represent the same tissue and were obtained from three different points on the corresponding tissue. (B) Images show the samples treated under low-temperature conditions. Numbering is the same as that for samples shown in panel (A). The green areas in the microphotographs show troponin T (scale bar, 50 μm). (C) Troponin-T-positive areas were quantified by image analysis [n = 3]. Error bars represent the SD. The *t-*test shows no significant difference.


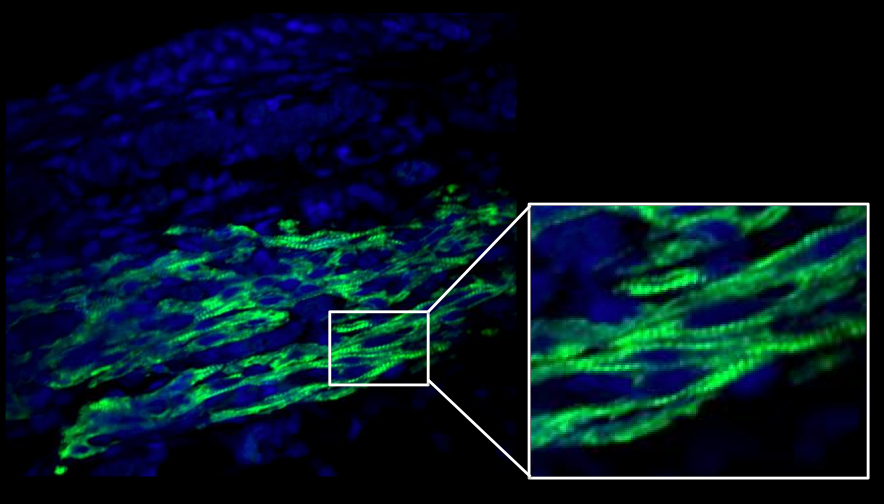


A

Low-temperature treated


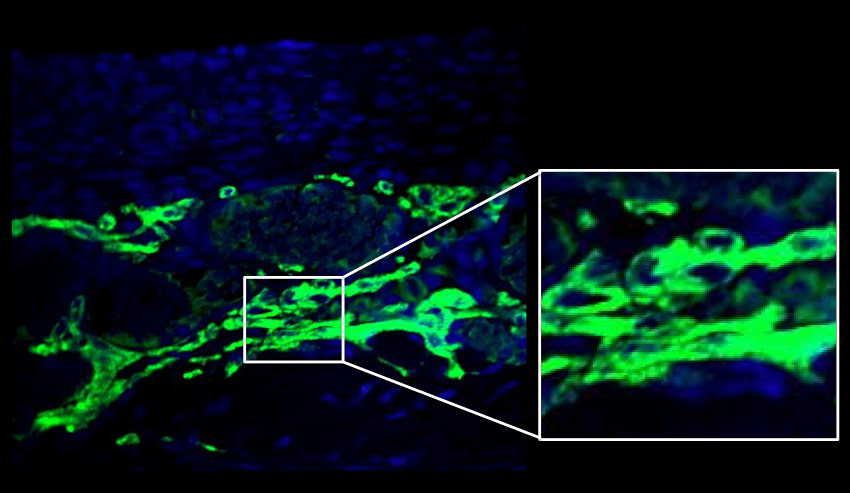


B

Non-treated

Figure S8. Troponin-stained specimens of a quintuple-layered cardiac cell sheet transplanted into a nude rat. The green and blue areas in the microphotographs show troponin T and nuclei, respectively. (A) A quintuple-layered cell sheet was cultured in a 30°C incubator for 7 days to stimulate vascularization and maintain the cardiomyocytes within the cell sheet. The low-temperature-treated cell sheet was transplanted into a nude rat. After 2 weeks, the cell sheet specimens were prepared and stained with troponin T. The white arrowheads indicate sarcomere formation in the transplanted cardiac cell sheet (scale bar, 50 μm). In contrast, photograph (B) shows a specimen of the transplanted quintuple-layered cell sheet prepared without the low-temperature treatment. The red arrowheads indicate thrombi among the cell layers. The thrombi hindered the cell sheet from attaching to the surface of the host and prevented cardiomyocytes from developing (scale bar, 50 μm).


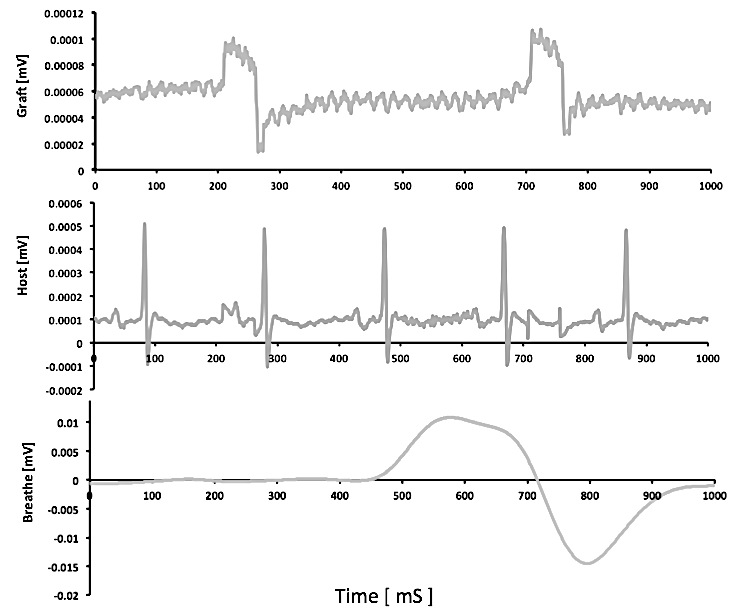


Graft beat

Host beat

Host breath

Figure S9. Electric potentials of the transplanted cell sheets. Potential differences that originated from the host’s breathing and heartbeat were observed.
